# FTO suppresses cardiac fibrosis after myocardial infarction via m^6^A-mediated epigenetic modification of EPRS

**DOI:** 10.1186/s10020-024-00985-7

**Published:** 2024-11-13

**Authors:** Jian Wang, Yanyan Li, Lijie Deng, Yafang Zha, Song Zhang

**Affiliations:** 1 Department of Emergency, Renji Hospital, Affiliated Shanghai Jiao Tong University School of Medicine, Shanghai, 200127 People’s Republic of China; 2 Department of Cardiology, Xinhua Hospital, Affiliated Shanghai Jiao Tong University School of Medicine, Shanghai, 200092 People’s Republic of China

**Keywords:** N^6^-methyladenosine (m^6^A), FTO, EPRS, Cardiac fibrosis

## Abstract

**Background:**

Cardiac fibrosis is common in myocardial infarction (MI), leading to progressive cardiac dysfunction. Studies suggested that the abnormal *N*^6^-methyladenosine (m^6^A) modification induced by fat mass and obesity protein (FTO) is vital in MI. However, the effects of FTO on post-infarction cardiac fibrosis have not been detected.

**Methods:**

Western blot and quantitative real-time PCR were performed to detect the expression of FTO in the fibrotic tissue of rats. The functions of FTO on collagen biosynthesis were analyzed in vitro and in vivo. The underlying targets of FTO were selected through RNA-seq with m^6^A-seq. The following dual luciferase reporter assay and RNA stability assay were conducted to investigate the mechanisms of FTO-mediated m^6^A regulation.

**Results:**

The expression of FTO was decreased in the fibrotic tissue of post-infarction rats. The HIF-1 signal pathway was enriched after MI. HIF-1α could bind to the promoter of FTO and inhibit its expression. Functionally, FTO inhibited collagen synthesis after MI in vitro and in vivo. Mechanistically, EPRS was selected as the underlying target of FTO-induced m^6^A regulation. IGF2BP3 recognized and bound to the m^6^A sites of EPRS mRNA, which improved its stability. EPRS was required for cardiac fibrosis induced by FTO silencing.

**Conclusions:**

FTO, identified as a cardioprotective factor, suppressed collagen synthesis in post-infarction cardiac fibrosis via m^6^A modification, which provided a new therapeutic strategy for cardiac fibrosis.

**Supplementary Information:**

The online version contains supplementary material available at 10.1186/s10020-024-00985-7.

## Introduction

Cardiac fibrosis is characterized by the abnormal proliferation of cardiac fibroblasts (CFs) and the excessive accumulation of various collagen-related proteins in the cardiac interstitium (Berk et al. 2007). Once myocytes are damaged, CFs become activated into myofibroblasts that migrate and produce collagens, including collagen I (COL-1), collagen III (COL-3), and other extracellular matrix components (Bacmeister et al. 2019). Increased deposition of collagen fibers leads to hypertrophic scarring, leading to impaired cardiac systole and diastole function. However, the specific molecular mechanisms underlying the development of cardiac fibrosis has not been elucidated. Therefore, it is necessary to explore new therapeutic targets for cardiac fibrosis.

Glutamyl-prolyl-tRNA synthetase (EPRS) is a protein that catalyzes the attachment of glutamic acid and proline to their cognate tRNAs for protein translation (Arif et al. 2018). Given that many fibrotic proteins, including collagen and IL-11, are proline-rich, EPRS-mediated translational regulation plays a crucial role in organ fibrosis (Keller et al. 2012; Song et al. 2018, 2019). Halofuginone (HF) has been reported to inhibit the activity of EPRS by blocking the binding of EPRS to proline and tRNA (Keller et al. 2012; Onuora 2023). However, it remains unknown whether modification at the nucleotide level can regulate the expression of EPRS, which could offer a new method of facilitating cardiac fibrosis.

*N*^6^-methyladenosine (m^6^A) is one of the most prevalent modifications in eukaryotic mRNAs. m^6^A refers to the methylation of the nitrogenous base at the sixth position of the adenosine in RNA (Dorn et al. 2019). m^6^A modification is processed by methylase (“writers”) containing Methyltransferase-like 14 (METTL14), Methyltransferase-like 3 (METTL3), and Methyltransferase-like 16 (METTL16) (Wang et al. 2016). The modification can be reversed by demethylase (“erasers”) including fat body mass and obesity-associated protein (FTO), and a-ketoglutarate-dependent dioxygenase homology 5 (ALKBH5) (Fu et al. 2013). In addition, RNA-binding proteins (“readers”) regulate the functions of RNA with m^6^A marks containing YT521-B homology domain-containing family proteins (YTHDF1-3), YTH domain-containing proteins (YTHDC1-2), and insulin-like growth factor 2 binding proteins (IGF2BP1-3) (Huang et al. 2018). Emerging evidence has shown that m^6^A regulation is strongly associated with cardiovascular diseases (Xu et al. 2020; Du et al. 2021; Ke et al. 2022). Nevertheless, the underlying mechanisms of cardiac fibrosis and m^6^A modification have not been fully explored.

The current study has explored the m^6^A modification in cardiac fibrosis induced by myocardial infarction (MI). Both FTO and EPRS were detected to be critical for cardiac fibrosis. EPRS was regulated by FTO in m^6^A-dependent mechanisms. This study may propose a new insight into the epigenetic regulation of cardiac fibrosis.

## Materials and methods

### Cell culture

Healthy Sprague-Dawley rats, within 3 days of birth, were provided by Jihui Laboratory Animal Breeding Co., Ltd. (Shanghai, China). Briefly, the hearts were harvested and digested with 0.125% trypsin (Gibco, USA) and 0.1% collagenase I (100 units/ml; Worthington, USA). The myocardial cells were purified by differential adhesion selection. Neonatal rat cardiac fibroblasts (NRCFs) were cultured in Dulbecco’s Modified Eagle Medium (DMEM) (Hyclone, USA) with 10% fetal bovine serum (FBS) (Yeasen, China) and penicillin-streptomycin (100 µg/mL, Beyotime, China). The cells were incubated at 37 ℃ with 95% O_2_ and 5% CO_2_. After 24 h, NRCFs were placed in a hypoxia chamber with 1% O_2_, 5% CO_2,_ and balanced with N_2_.

### Myocardial infarction model

Male healthy Sprague-Dawley rats (weighing 160–200 g, 5 weeks old) were purchased from Jihui Laboratory Animal Breeding Co., Ltd. (Shanghai, China). Rats were intravenously injected with an adenovirus containing FTO or an empty vector (1 × 10^9^ vector genomes particles per rat) (Heyuan Biotechnology, China) 1 week before the MI operation. Animals were anesthetized with 3% sodium pentobarbital (50 mg/Kg) and ventilated with an animal ventilator. The model of MI was established via permanent ligation of the left anterior descending coronary artery (LAD) with a 5/0 nylon suture. The sham rats went through all the procedures without the ligation of the LAD. The rats were anesthetized for echocardiography and sacrificed 4 weeks after MI.

### Transcriptome sequencing (RNA-seq) and methylated RNA immunoprecipitation sequencing (m6A-seq)

NRCFs were transfected with an FTO plasmid. Cells were collected by TRIzol (Takara, Japan) after transfection of 48 h. m^6^A-seq was performed by Cloud-Seq Biotech (Shanghai, China). RNA was subjected to immunoprecipitation with the GenSeq^®^ m6A MeRIP Kit (GenSeq, China) by following the manufacturer’s instructions. The m^6^A-IP and input samples were then used to construct for the RNA-seq library with GenSeq^®^ Low Input Whole RNA Library Prep Kit (GenSeq, China) following the manufacturer’s instructions. The libraries were sequenced on a NovaSeq platform (Illumina). Transcriptome sequencing was performed by JiayinTech (Shanghai, China). The different gene expressions were detected on the Illumina sequencing platform. The statistically different genes were identified by a log_2_ fold change ≥ 1 and a *p* value < 0.05.

### RNA dot blot

Total RNA was isolated from NRCFs and rat heart tissue. Total RNA samples (250ng and 100ng respectively) were spotted on the nylon membrane (Beyotime, China). The membrane was then ultraviolet (UV) crosslinked at 125 mJoule/cm^2^. The membranes were incubated with anti-m^6^A antibody (SySy, USA) overnight at 4℃ after being blocked in 5% non-fat milk for 1 h. The membranes were washed and incubated with a secondary anti-rabbit polyclonal antibody (Beyotime, China). Signals were revealed by enhanced chemiluminescence ECL (Thermo Scientific) with an image capture system (Tanon 5200) and quantified by densitometry software (Image-Pro Plus). Subsequently, the membranes were stained with methylene blue (Sigma) for 2 h and washed with RNA-free water for 5 min.

### CUT&Tag - Seq

The NovoNGS^®^ CUT&Tag 4.0 High-Sensitivity Kit was provided by Novoprotein Scientific Inc. Briefly, the cells (about 10^5^/cells) were bound to ConA magnetic beads. Then Digitonin was added to penetrate the cell membrane, and the primary anti-HIF1α antibody (Abcam, USA), ChiTag goat anti-rabbit IgG antibody, and pAG-Tn5 were desiccated and segmented successively. The reaction products were extracted by DNA Clean Beads and enriched by PCR to construct the second-generation sequencing library. The libraries were sequenced on an Illumina NovaSeq 6000 for the generation of 150-bp paired-end reads.

### Supplementary methods

The rest of methods supporting the conclusions of this article were included within the supplementary materials.

### Statistical analysis

Data are presented as mean ± standard error (SEM). All statistical analyses were performed by GraphPad Prism software (GraphPad software version 8.0; GraphPad Inc, San Diego, CA, USA). Differences between two groups or multiple groups were analyzed respectively by Student’s *t* test or ANOVA. A *p-value* < 0.05 was considered statistically significant.

## Results

### FTO expression was decreased in cardiac fibrotic tissue and in hypoxia-induced fibroblasts, which accounted for the excessive m^6^A modification

**Fig. 1:**
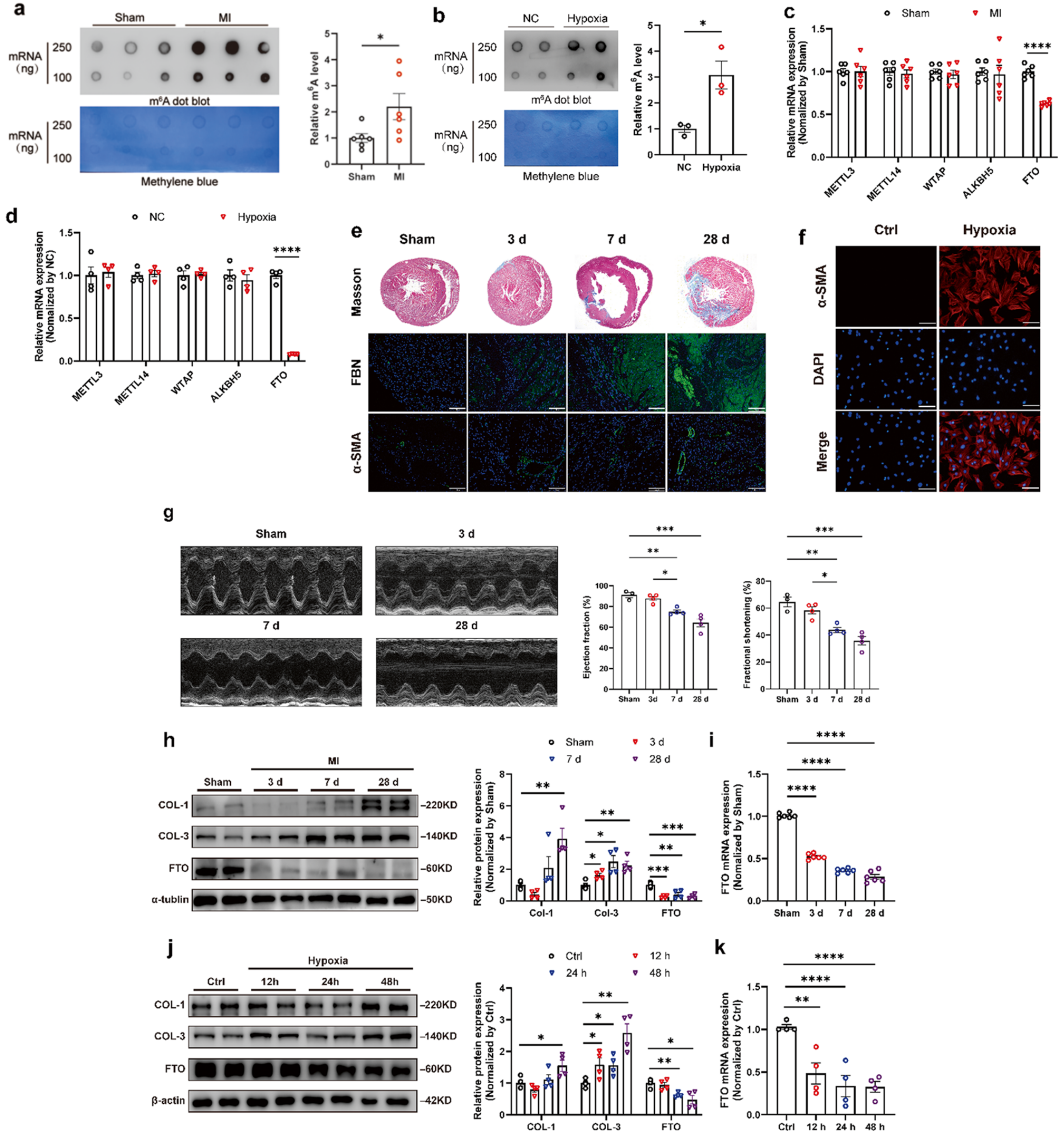
Demethylase FTO expression decreased in rat fibrotic heart tissue and hypoxia-mediated cardiac fibroblasts. RNA dot blot of m^6^A levels in MI rats (**a**) and hypoxia-induced CFs (**b**). The methylene blue staining served as an internal control. The mRNA expression of m^6^A-regulation-related genes in MI (**c**) and hypoxia-treated CFs (**d**) respectively. **e** Representative Masson’s trichrome and immunofluorescence images of hearts after MI. Scale bar 100 μm. **f** Representative histological images with immunofluorescence staining of CFs under hypoxia conditions. Scale bar 20 μm. **g** Representative M-mode images of MI-treated rats showing ejection fraction (EF) and fraction shortening (FS) evaluated by echocardiography. *n* = 3–4. **h** Expression of Collagen 1 (COL-1), Collagen 3 (COL-3), and FTO in a rat model of cardiac fibrosis 3, 7, and 28 days after MI. *n* = 4. **j** Expression of COL-1, COL-3, and FTO in a cellular model of fibrogenesis induced by hypoxia for 12, 24, and 48 h. *n* = 4. Representative FTO expression was determined by qRT-PCR in rat (**i**) and cellular (**k**) fibrosis models at different intervention times. *n* = 4. The data was expressed as mean ± SEM. ^*^P < 0.05 vs. Ctrl/Sham, ^**^P < 0.01 vs. Ctrl/Sham, ^****^P < 0.0001 vs. Ctrl/Sham

To understand the role of m^6^A modification in cardiac fibrosis, we assessed the expression under cardiac fibrosis in vivo and in vitro. An RNA m^6^A dot blot was performed to measure the levels of m^6^A modification. The results revealed that the m^6^A levels were significantly elevated in the cardiac fibrotic tissue of rats (Fig. 1a). The biological process of MI was accompanied by hypoxia. Subsequently, we established the hypoxia chamber (1% O_2_, 5% CO_2,_ and balanced with N_2_) to explore the activation of CFs. Then we measured the m^6^A levels in CFs under hypoxia treatment. Consistent with the expression levels in vivo, hypoxia promoted m^6^A modification of CFs (Fig. 1b). The above results demonstrated that MI or hypoxia upregulated the levels of m^6^A modification in rats or CFs respectively. Evidence has confirmed that m^6^A modification levels were adjusted by methylases (METTL3, METTL14, WTAP) and demethylases (FTO, ALKBH5)(Meyer and Jaffrey 2017; Roignant and Soller 2017). To find which methylation-related enzyme caused the abnormal m^6^A modification, the expression levels of these m^6^A-associated genes were measured in MI rats and hypoxia-treated CFs (Fig. 1c, d). FTO was decreased in both MI tissues and hypoxia-induced CFs. However, the expression of METTL3, METTL14, WTAP, and ALKBH5 remained unchanged (Fig. 1c, d).

To further know the role of aberrant FTO expression in cardiac fibrosis, the cardiac fibrosis rat model was established by ligation of LAD for 3, 7, and 28 days, and the hypoxia-induced CFs model was carried out in the hypoxia chamber for 12, 24, and 48 h. It was observed that the cardiac function of MI rats significantly decreased at 7 days post-surgery and fibrotic markers, containing fibronectin (FBN) and α-smooth muscle actin (α-SMA), increased along with MI progression (Fig. 1e). Consistent with the above results in vivo, α-SMA was markedly stimulated by hypoxia for 48 h (Fig. 1f). Western blot and quantitative real-time PCR (qRT-PCR) analysis measured the negative relationship between FTO and fibrotic markers in both the fibrotic tissue of rats and hypoxia-induced CFs, including COL-1 and COL-3 (Fig. 1h, j). In the fibrotic tissue of rats, FTO expression was significantly decreased at both the mRNA and protein levels 3 days post-MI surgery (Fig. 1h, i). Similarly, FTO expression was downregulated in hypoxia-induced CFs for 24 h at both mRNA and protein levels (Fig. 1j, k).

### HIF1α bound to FTO promoter and decreased FTO expression

**Fig. 2:**
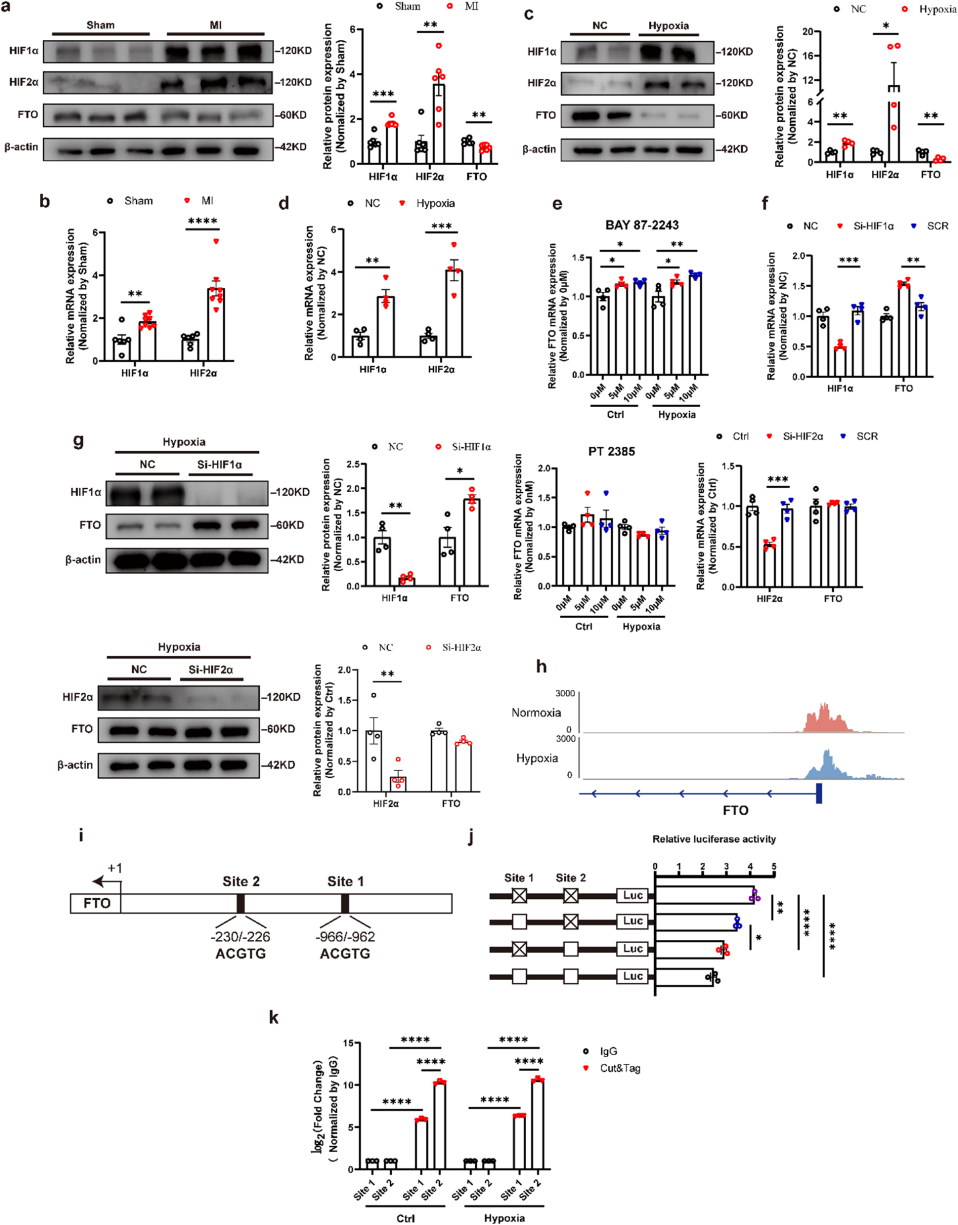
MI-induced HIF1α accumulation negatively regulated FTO by binding to its promoter. Western blot (**a**) and qRT-PCR (**b**) results of HIF1α, HIF2α, and FTO in MI heart tissues. *n* = 6. The protein (**c**) and mRNA (**d**) levels of HIF1α, HIF2α, and FTO in hypoxia-treated cardiac fibroblasts (CFs). *n* = 4. **e** Expression level of FTO after treatment of HIF1α inhibitor BAY 87-2243 and HIF2α inhibitor PT2385. *n* = 4. Western blot (**g**) and qRT-PCR (**f**) results showing the expression of HIF1α, HIF2α, and FTO after HIF1α and HIF2α knockdown in CFs. *n* = 4. **h** Representative CUT&Tag-seq signal tracks at the promoter region of FTO. **i** CUT&Tag-seq predicting the potential binding sites of HIF1α and FTO promoter. **j** Relative luciferase activity after transfection with reporter plasmids showing the binding sites. The ratio of Firefly and Renilla luciferase values calculated the relative luciferase activity. **k** The CUT&Tag-qPCR results showing the degree of enrichment in predicted binding sites of the FTO promoter. *n* = 3. SCR, scramble sequences group. NC, negative control. The data was expressed as mean ± SEM. ^*^P < 0.05 vs. NC/SCR, ^**^P < 0.01 vs. NC/SCR, ^***^P < 0.001 vs. NC/SCR, ^****^P < 0.0001 vs. NC/SCR

The biological process of MI was accompanied by hypoxia. It was widely known that the HIF1 signal pathway was highly activated under hypoxic conditions (Ikeda et al. 2021; Sato and Takeda 2023). Thus, we detected the expression levels of the HIF signal pathway in MI rats and hypoxia-cultured CFs. Western blot and qRT-PCR results showed that HIF1α and HIF2α levels increased as FTO levels decreased in MI rat models (Fig. 2a, b). Then, hypoxia accelerated the protein and mRNA levels of HIF1α and HIF2α while inhibiting the expression of FTO (Fig. 2c, d). To identify the biological roles and molecular mechanisms of HIF1α and HIF2α, the specific inhibitors and knockdown of HIF1α and HIF2α were performed. BAY 87-2243, a HIF1α inhibitor, significantly promoted FTO mRNA levels by contrast with PT2385, a HIF2α inhibitor, remaining FTO unchanged (Fig. 2e). Then, the knockdown of HIF1α and HIF2α of CFs were established. The silencing efficacy at both mRNA and protein levels was confirmed (Fig. 2f, g). However, only HIF1α knockdown increased the FTO expression level (Fig. 2f, g). Therefore, HIF1α, rather than HIF2α, negatively regulated FTO expression.

A CUT&Tag assay was performed to elucidate the specific molecular mechanisms by which HIF1α regulated FTO expression. The results revealed that the binding sites of HIF1α were primarily located in the transcriptional start site (TSS) under normoxia and hypoxia conditions (Fig. S1a, b, c). Signal pathways correlated with DNA replication and CF activation were enriched in HIF1α-mediated CUT&Tag samples (Fig. S1d). The CUT&Tag data displayed peaks at the FTO promoter, suggesting the specific binding sites of HIF1α (Fig. 2h). Abundant evidence has confirmed that HIF-1α could bind to the promoter of target genes via hypoxia transcriptional response elements (HRE) (Liu et al. 2018; Singh et al. 2022). Then, we analyzed the sequences and found two potential HRE sites in the FTO promoter (Fig. 2i). Initially, we conducted a luciferase reporter plasmid controlled by the rat FTO promoter (-1034 bp upstream of TSS, FTO-luc). To identify the importance of these HRE sites for HIF1α-regulated FTO expression, site-directed mutagenesis was carried out to mutate the HRE motifs from ACGTG to AAGGA in the FTO-luc construct. Mutations at Site 1 and Site 2 significantly increased the HIF1α-regulated FTO expression, although Site 1 had only minor effects (Fig. 2j). The following CUT&Tag-qPCR experiments also confirmed greater enrichment at Site 2 compared to Site 1 (Fig. 2k).

In all, the above data demonstrated that HIF1α bound to the FTO promoter via HRE in CFs. MI or hypoxia treatment activated HIF1α and inhibited gene *FTO* transcription. Thus, HIF1α is a transcriptional inhibitor of FTO.

### FTO regulated collagen production and proliferation of CFs in vitro

**Fig. 3:**
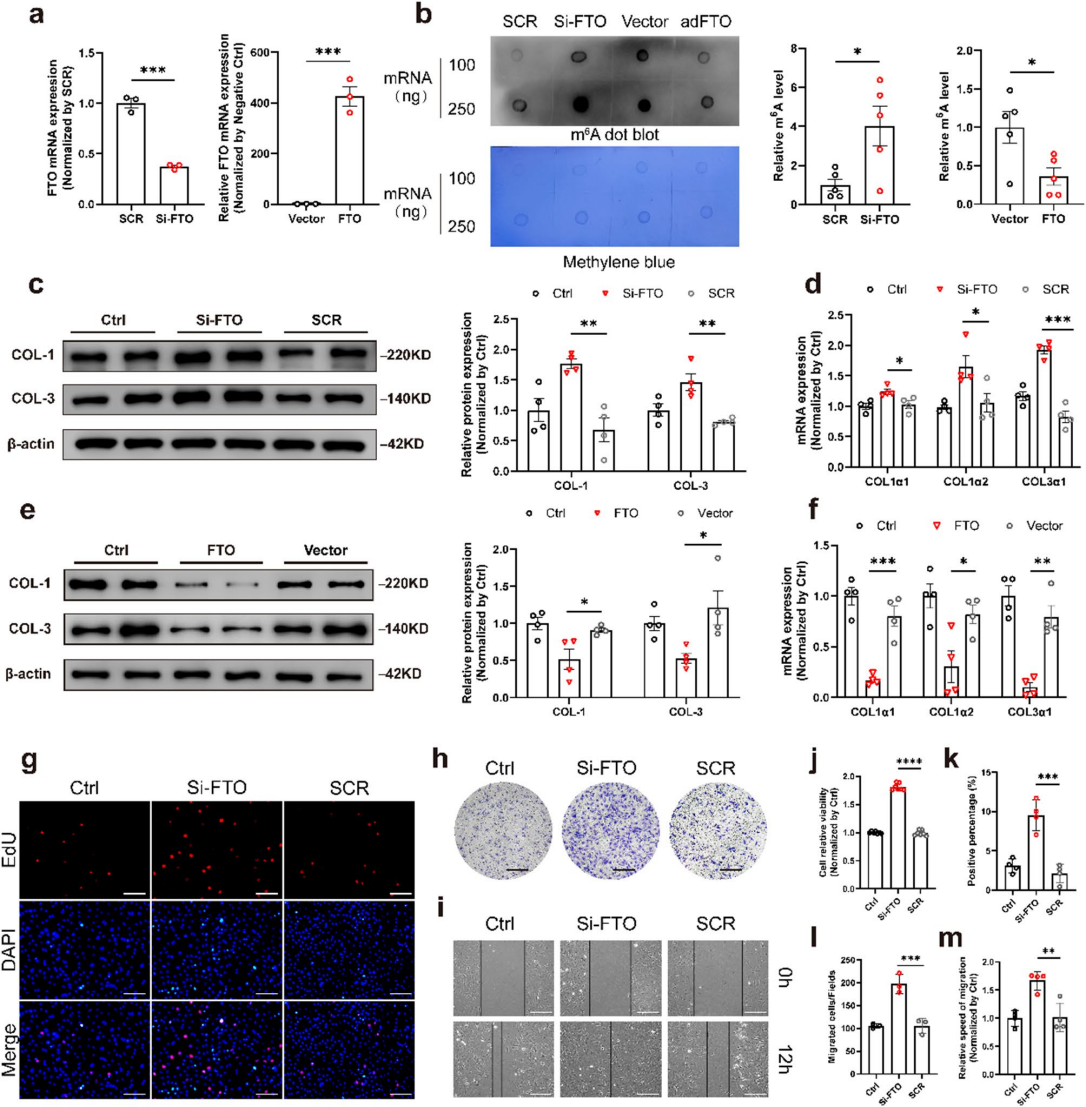
FTO expression modulated collagen production and fibroblast activation in cardiac fibroblasts. **a** RNA dot blot of m^6^A levels in cardiac fibroblasts (CFs) with transfection of Si-FTO and FTO overexpression plasmids (FTO). The methylene blue staining served as a loading control. *n* = 5. **b** The relative FTO mRNA levels of CFs after transfection of Si-FTO and FTO. *n* = 3. The protein (**c**) and mRNA (**d**) expression of Collagen 1 (COL-1) and Collagen 3 (COL-3) after transfection of si-FTO. *n* = 4. The protein (**e**) and mRNA (**f**) levels of COL-1 and COL-3 after transfection of FTO. *n* = 4. **g**,** k** The EdU fluorescence dying assay after transfection of Si-FTO. Scale bar 50 μm. *n* = 4. Representative images (**h**) and analysis (**l**) of transwell assay captured at 12 h after transfection of Si-FTO. Scale bar 100 μm. *n* = 4. Microscope images (**i**) of wound healing assay captured at 0 and 24 h after transfection of Si-FTO. The relative speed of migration is shown in (**m**). Scale bar 100 μm. *n* = 4. **j** Cell viability after transfection of Si-FTO. *n* = 7. SCR, scramble sequences group. Vector, empty plasmid. The data was expressed as mean ± SEM. ^*^P < 0.05 vs. SCR/Vector, ^**^P < 0.01 vs. SCR/Vector, ^***^P < 0.001 vs. SCR/Vector, ^****^P < 0.0001 vs. SCR/Vector

To determine the function of FTO in CFs, we performed gain- and loss-of-function experiments by transfecting CFs of small interfering RNA targeting FTO (Si-FTO) and an overexpression plasmid of FTO (FTO). The transfection efficiencies of Si-FTO and FTO were validated by qRT-PCR (Fig. 3a). An RNA m^6^A dot blot assay was performed to measure the levels of m^6^A modification, which showed that Si-FTO increased the m^6^A levels while FTO decreased the m^6^A levels in CFs (Fig. 3b). The mRNA (Fig. 3d) and protein (Fig. 3c) levels of COL-1 and COL-3 were markedly increased after transfection of Si-FTO (100nM) under normal conditions. On the contrary, western blot and qRT-PCR analyses indicated decreased expression levels of COL-1 and COL-3 following transfection with FTO (1 µg/ml) without any treatment (Fig. 3e, f). 5-ethynyl-2’ -deoxyuridine (EdU) fluorescence staining showed that FTO knockdown significantly promoted the proliferation of CFs (Fig. 3g, k), and similar results were obtained from the CCK-8 assay (Fig. 3j). Meanwhile, compared with the scramble sequence group (SCR), CFs treated with Si-FTO markedly increased fibroblast migration indexed by the Transwell assay (Fig. 3h, l) and wound healing assay (Fig. 3i, m).

### Protective effects of FTO on MI-induced cardiac fibrosis

**Fig. 4:**
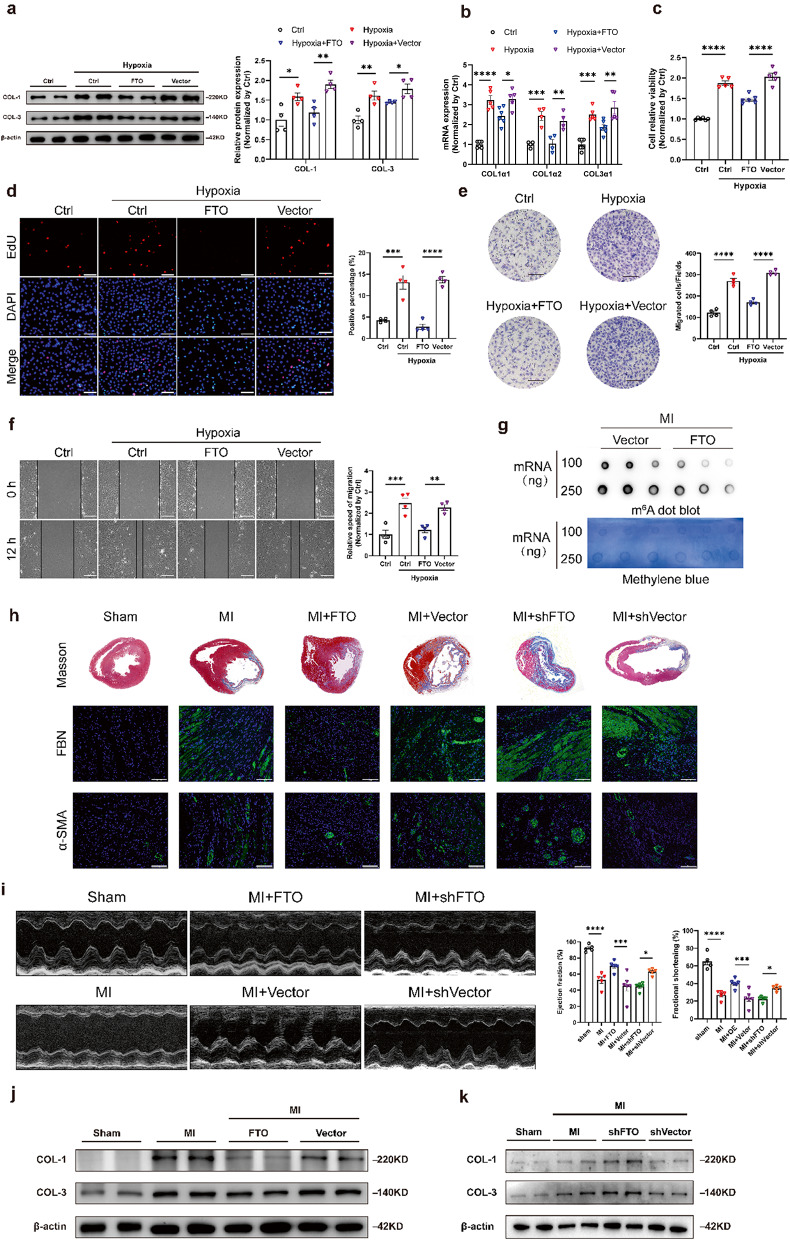
Effects of FTO overexpression and knockdown on hypoxia-mediated fibrosis and MI-induced cardiac fibrosis. The protein (**a**) and mRNA (**b**) levels of Collagen 1 (COL-1) and Collagen 3 (COL-3) after transfection of FTO overexpression plasmid (FTO) in cardiac fibroblasts (CFs) under hypoxia stimulation. *n* = 4. **c** Cell viability with transfection of FTO in hypoxia-induced CFs. *n* = 5–6. **d** The EdU fluorescence dying assay after transfection of FTO. Scale bar 50 μm. *n* = 4. **e** Representative images and quantitative analysis of transwell assay captured at 12 h accompanied by transfection of FTO. Scale bar 100 μm. *n* = 4. **f** Representative images of wound healing assay captured at 0 and 24 h after transfection of FTO. Scale bar 100 μm. *n* = 4. **g** RNA dot blot of m^6^A levels in MI rats with FTO-overexpressing virus. The methylene blue staining served as an internal control. **h** Representative histological images with Masson’s trichrome and immunofluorescence staining of MI heart. Scar bar 100 μm. **i** Representative M-mode images of MI-treated rats after overexpression of FTO and knockdown of FTO showing ejection fraction (EF) and fraction shortening (FS) evaluated by echocardiography. *n* = 5–6. The protein levels of COL-1 and COL-3 in fibrotic heart tissue induced by MI with FTO overexpression (**j**) and FTO silencing (**k**). Vector/shVector, the empty virus vector. The data was expressed as mean ± SEM. ^*^P < 0.05 vs. Sham/Vector, ^**^P < 0.01 vs. Sham/Vector, ^***^P < 0.001 vs. Sham/Vector. ^****^P < 0.0001 vs. Sham/Vector

Since the above findings indicated that FTO inhibited collagen synthesis under normal conditions, we detected the protective effects of FTO under pathological conditions. We first evaluated the protective effects of FTO on hypoxia-induced CFs. The results indicated that hypoxia stimulation increased collagen expression at both mRNA and protein levels. Consistent with the findings in normal conditions, overexpression of FTO reduced the mRNA and protein levels of collagen (Fig. 4a, b). In addition, FTO overexpression reduced hypoxia-mediated cell proliferation, as determined by the CCK-8 assay (Fig. 4c) and EdU fluorescence staining (Fig. 4d). Similarly, the migration ability of CFs was weakened as shown by the Transwell assay (Fig. 4e) and wound healing assay (Fig. 4f).

Then, we constructed an adenovirus carrying FTO to investigate the effects on MI rat models. The efficiency of adenovirus infection was confirmed (Fig. S2). After injection of FTO-overexpressing adenovirus for 7 days, the MI model was established by ligation of LAD. Interstitial fibrosis in MI rats was evaluated 4 weeks post-MI surgery. m^6^A levels in fibrotic tissues were significantly downregulated after FTO overexpression treatment (Fig. 4g). In addition, the lentivirus carrying shFTO was conducted for further detection. We observed that FTO overexpression therapy reduced MI-activated fibrotic markers, including collagen deposition, FBN, and α-SMA in contrast with FTO silencing accelerating fibrotic markers (Fig. 4h). Echocardiography was performed to assess the protective effects of FTO on cardiac function. Compared with the vector group, FTO overexpression markedly ameliorated the cardiac function of MI rats and collagen deposition (Fig. 4i, j). Conversely, the cardiac function and collagen production of MI rats with FTO knockdown significantly deteriorated (Fig. 4i, k).

Collectively, these results manifested that FTO inhibited collagen biosynthesis and activation of CFs. overexpressed FTO could reduce collagen deposition and ameliorate the cardiac function of post-MI rats.

### FTO targeted EPRS in m^6^A-seq combined with RNA-seq

**Fig. 5:**
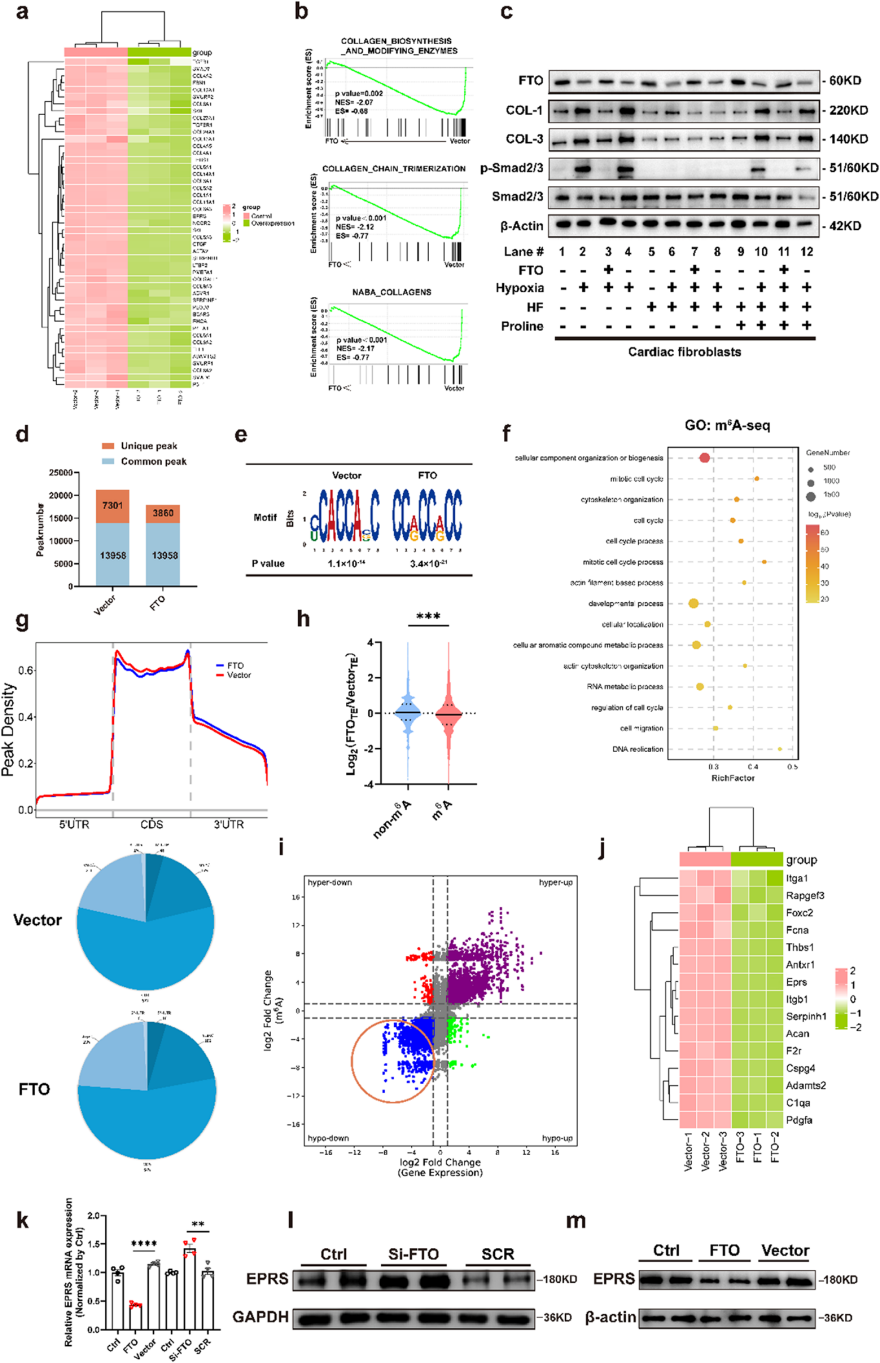
EPRS is identified as the downstream target of FTO via m^6^A modification. **a** Differentially expressed genes associated with collagen biosynthesis between Vector and FTO group in CFs (red represented up-regulation and green represented down-regulation). **b** Differentially gene profiles based on GSEA of FTO and Vector RNA-seq data. **c** Control or FTO-overexpressing CFs were treated with hypoxia, HF (100 nM), and/or proline (2 mM) for 24 h before preparation of whole-cell extracts for immunoblotting. **d** Number of unique and common m^6^A peaks in CFs of Vector and FTO groups. **e** Motif detected “CCACC” as the m^6^A consensus motif of CFs. **f** Down-regulated enrichment maps of biological process from m^6^A-seq reads in CFs. **g** Distribution of m^6^A peaks in mRNA transcripts in CFs. **h** Distribution of gene foldchange in RNA-seq with or without m^6^A modification. **i** The star plot revealing the distribution of genes with both differential (hyper or hypo) m^6^A peaks (Y-axis: fold change > ± 1, *P* < 0.05) and differential (up or down) expression (X-axis: fold change > ± 1, *P* < 0.05) in the FTO group compared with the vector group. The blue dots highlighted by an orange circle displaying down-regulated transcripts with decreased m^6^A levels were selected for the following investigations. **j** The collagen-associated genes with different m^6^A and mRNA expression levels shown in the heat map (red represented up-regulation and green represented down-regulation). **k** Relative mRNA expression of EPRS in Si-FTO- and FTO-treated CFs. *n* = 4. Western blot results showing the protein levels of EPRS in CFs with FTO knockdown (**l**) and FTO overexpression (**m**). *n* = 4. SCR, scramble sequences group. Vector, empty plasmid. The data was expressed as mean ± SEM. ^**^P < 0.01 vs. Sham/Vector, ^***^P < 0.001 vs. Sham/Vector. ^****^P < 0.0001 vs. Sham/Vector

RNA-seq was performed to investigate the underlying targets of FTO in cardiac fibrosis. Compared to the vector group, the results identified a total of 2929 differentially expressed genes (1547 upregulated genes, 1382 downregulated genes) in the FTO-overexpressed group. Overexpressed FTO resulted in decreased mRNA levels of collagen-related genes, including EPRS, Col1α1, Col3α1, and FBN (Fig. 5a). Furthermore, gene set enrichment analysis (GSEA) in RNA-seq indicated that collagen, collagen biosynthesis and modifying enzymes, and collagen chain trimerization were negatively enriched in the FTO-overexpressing CFs, suggesting the potential targets involved collagen synthesis (Fig. 5b). Proline is an essential amino acid for collagen production (Arif et al. 2018). HF, a febrifugine derivative, specifically inhibited collagen I synthesis and acted as a competitive inhibitor of prolyl-tRNA synthetase (Keller et al. 2012). However, the decreased expression of collagen I could be rescued by proline supplementation (Song et al. 2019). Then, the western blot was performed to elucidate the specific mechanisms of FTO-mediated fibrosis. The results indicated that HF treatment decreased hypoxia-induced and FTO-dependent collagen expression, which could be reversed with supplemental proline therapy (Fig. 5c). These findings revealed that FTO had a distinct role in proline-mediated collagen production. Moreover, Gene Ontology (GO) analysis of biological process suggested that FTO overexpression significantly suppressed cell migration and cell proliferation (Fig. S3a). Based on Kyoto Encyclopedia of Genes and Genomes (KEGG) analysis, the MAPK, PI3K-Akt, and Wnt signaling pathways, which were closely associated with the activation of CFs, were negatively enriched in FTO-overexpressed CFs (Fig. S3b). In all, FTO suppressed the activity of CFs according to RNA-seq data.

To verify whether genes involved in collagen synthesis were differentially regulated by FTO-mediated m^6^A modification, m^6^A-seq was performed. There were 21,259 and 17,818 m^6^A peaks identified by m^6^A-seq, of which 7301 and 3860 were unique in the vector and FTO-overexpressed groups respectively (Fig. 5d). Then, the m^6^A consensus motif CCACC was highly enriched in the m^6^A peaks (Fig. 5e). Consistent with previous studies, m^6^A modifications were mainly enriched around the initiation and stop codon of the coding region (CDS) in Vector and FTO groups (Fig. 5g). GO analysis of biological process was also performed and indicated that FTO reduced cell cycle progression and cell migration in an m^6^A manner, which was consistent with RNA-seq data (Fig. 5f). Furthermore, KEGG analysis suggested that FTO overexpression remarkably reduced signaling pathways correlated with DNA replication and cell cycle (Fig. S3c). The above sequencing data confirmed that FTO regulated the activation of CFs via m^6^A modification. Subsequently, m^6^A-seq and RNA-seq were combined to find out the precise underlying targets of FTO-induced fibrosis. Genes were divided into the m^6^A group and the non-m^6^A group, depending on whether they were regulated by FTO-induced m^6^A modification. The results showed that the transcriptome fold change of genes in the m^6^A group was significantly lower and greater than that in the non-m^6^A group (Fig. 5h). This result suggested that FTO might downregulate the underlying gene expression levels via reducing m^6^A modification. Therefore, we focused on the decreased mRNA expression of genes with decreased m^6^A modification levels, which were highlighted in an orange circle (Fig. 5i). Among this circle, m^6^A-seq uncovered 3703 differential m^6^A peaks with decreased abundance in the FTO overexpressing group. Meanwhile, RNA-seq data identified corresponding 900 downregulated transcripts in FTO overexpression (Fig. 5i). Based on the above results and GO analysis (Collagen fibril organization), we focused on the collagen-related genes of which both m^6^A modification and expression levels were decreased. The above results suggested that FTO regulated collagen biosynthesis in a proline-dependent manner (Fig. 5c). Then, we screened out 15 genes involved in collagen synthesis from the overlap and discovered that EPRS may be the potential target of FTO-mediated m^6^A regulation due to promoting collagen translation in CFs since it was a protein that catalyzed the attachment of glutamic acid and proline to their cognate tRNAs for protein translation (Fig. 5j). qRT-PCR (Fig. 5k) and western blot (Fig. 5l, m) analysis verified that ERPS was negatively correlated with FTO.

### FTO regulated EPRS mRNA stability in m^6^A-dependent manners mediated by IGF2BP3

**Fig. 6:**
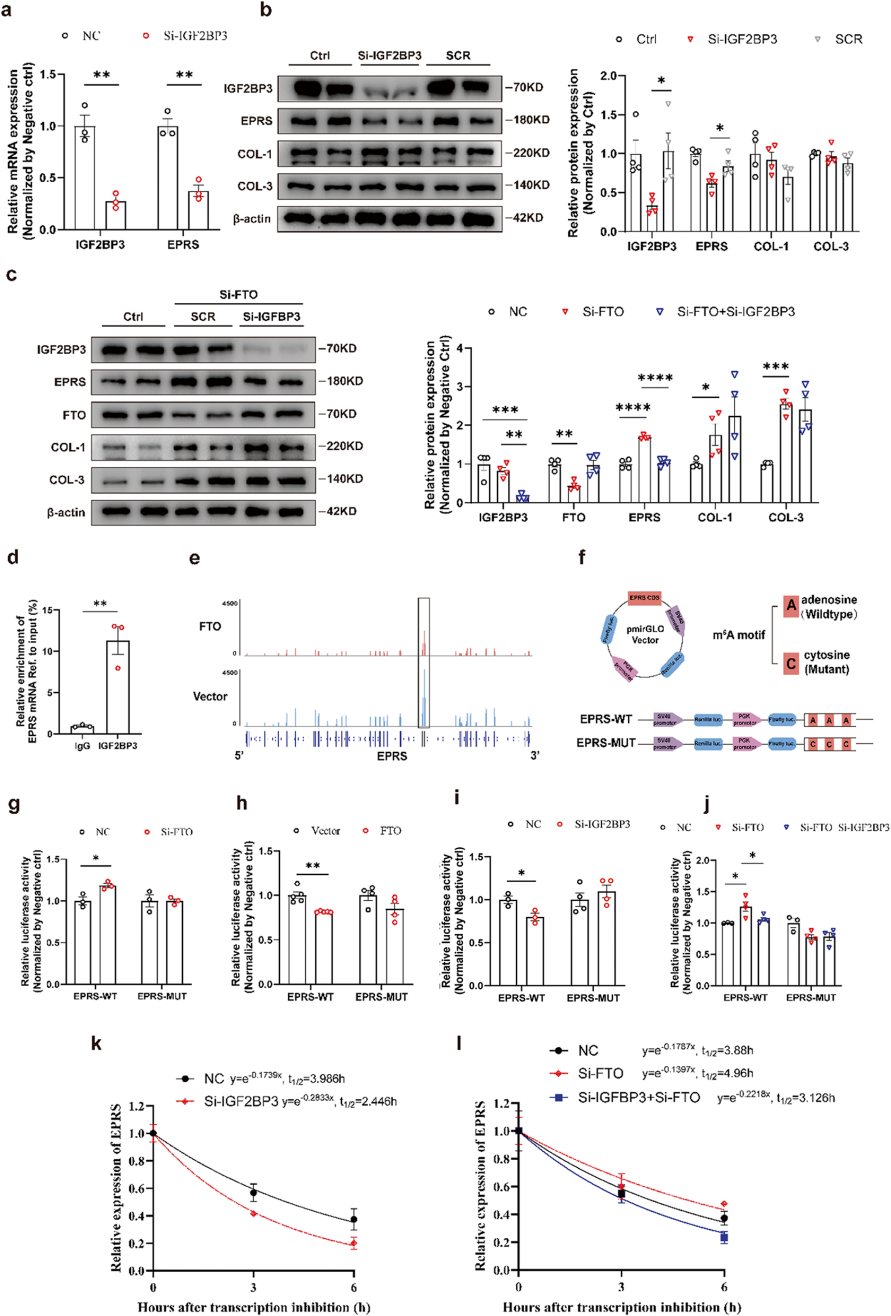
Stability of EPRS mRNA impaired by FTO-induced m^6^A modification via m^6^A reader IGF2BP3. **a** Relative expression of EPRS and IGF2BP3 mRNA after knockdown of IGF2BP3 in cardiac fibroblasts (CFs). *n* = 3. **b** The protein expressions of IGF2BP3, EPRS, Collagen 1 (COL-1), and Collagen 3 (COL-3) after transfection with IGF2BP3 knockdown in CFs. *n* = 4. **c** Western blot results of IGF2BP3, EPRS, FTO, COL-1, and COL-3 in CFs (control or FTO knockdown), with the absence or presence of IGF2BP3 silencing. *n* = 4. **d** m^6^A abundance of EPRS mRNA in cardiac fibroblasts between vector and FTO groups plotted by Integrative Genomics Viewer (IGV). **e** Graphical explanation for luciferase reporters showing that the wild type (full-length) or mutant (m^6^A motif mutated) sequence of the EPRS CDS region was inserted into the pmirGLO vector. **f** Relative enrichment of EPRS mRNA with IGF2BP3. The IgG group as a negative control for unspecific binding. Y-axis displaying the percentage of input for each IP sample. *n* = 3. Relative luciferase activity after transfection of EPRS-wild type or EPRS-mutated, with knockdown (**g**) or excessive (**h**) expression of FTO, and silencing of IGF2BP3 (**i**). *n* = 3 for **g** and *n* = 4–5 for **h**. **j** The ratio of Firefly and Renilla luciferase values calculated the relative luciferase activity in CFs (control or FTO knockdown), with the absence or presence of IGF2BP3 silencing. *n* = 3. **k** Half-life (t_1/2_) of EPRS mRNA determined by qRT-PCR in FTO-knockdown cells with actinomycin D and harvested at 0, 3, and 6 h. The mRNA stability normalized to the expression at 0 h. *n* = 3. **l** Half-life (t_1/2_) of EPRS mRNA in CFs (control or FTO knockdown), with the absence or presence of IGF2BP3 silencing. *n* = 3. NC, the negative control. SCR, scramble sequences group. Vector, empty plasmid. The data was expressed as mean ± SEM. ^*^P < 0.05 vs. NC/ Vector/SCR, ^**^P < 0.01 vs. NC/ Vector/IgG, ^***^P < 0.001 vs. NC/SCR/Vector, ^****^P < 0.0001 vs. SCR/Vector

The m^6^A modification levels of the target were modified by methylase and demethylase. Then the biological effects of m^6^A regulation were dependent on selective recognition of m^6^A sites by “readers”. IGF2BP1-3 have been reported to stabilize the mRNA levels (Huang et al. 2018; Feng et al. 2021). To identify which “reader” regulated the stability of EPRS mRNA, knockdown experiments for IGF2BP1-3, YTHDC1-2, and YTHDF1-3 were conducted. Both western blot and qRT-PCR analysis showed that the knockdown of IGF2BP3, not IGF2BP1/2, significantly reduced EPRS at both mRNA and protein levels of CFs (Fig.S5; Fig. 6a, b). Then, we further detected whether EPRS expression in FTO-knockdown CFs was affected by IGF2BP3. Silencing of IGF2BP3 remarkably inhibited the protein level of EPRS in CFs with FTO knockdown (Fig. 6c). It was realized that EPRS was the target of IGF2BP3. The following RIP-qPCR experiment confirmed that EPRS mRNA interacted with IGF2BP3 (Fig. 6d).

The m^6^A -seq data revealed that the m^6^A peak of EPRS in CDS shrank significantly with FTO overexpression (Fig. 6e). To verify the vital role of m^6^A modification in regulating EPRS mRNA, a luciferase reporter assay was established using either a wild-type EPRS-CDS sequence (WT) or a mutant counterpart (MUT) with altered m^6^A sites (Fig. 6f). While FTO levels was reduced, the relative luciferase activity in CFs with EPRS-WT plasmid significantly increased, whereas there was no significant change in CFs with EPRS-MUT plasmid (Fig. 6g). On the contrary, when FTO was overexpressed, the relative luciferase activity with EPRS-WT was downregulated (Fig. 6h). Moreover, the dual luciferase reporter assays demonstrated that knockdown of IGF2BP3 inhibited the luciferase activity of ERPS-WT plasmid through recognition of m^6^A sites (Fig. 6i, j). These findings suggested that IGF2BP3 regulated the expression level of EPRS through m^6^A modification. Next, RNA stability assays were conducted, and the results showed that IGF2BP3 deficiency promoted the degradation of EPRS mRNA (Fig. 6k). RNA degradation curves suggested that the half-life of EPRS mRNA was prolonged with FTO silencing in CFs, while IGF2BP3 knockdown could reverse the increased mRNA stability mediated by FTO silencing (Fig. 6l).

In summary, the upregulation of EPRS induced by FTO silencing can be attributed to the increased stability of EPRS mRNA, which was induced by elevated m^6^A modification. FTO regulated EPRS mRNA stability in m^6^A-dependent manners mediated by IGF2BP3.

### EPRS was required for SiFTO-promoted fibrosis in CFs

**Fig. 7:**
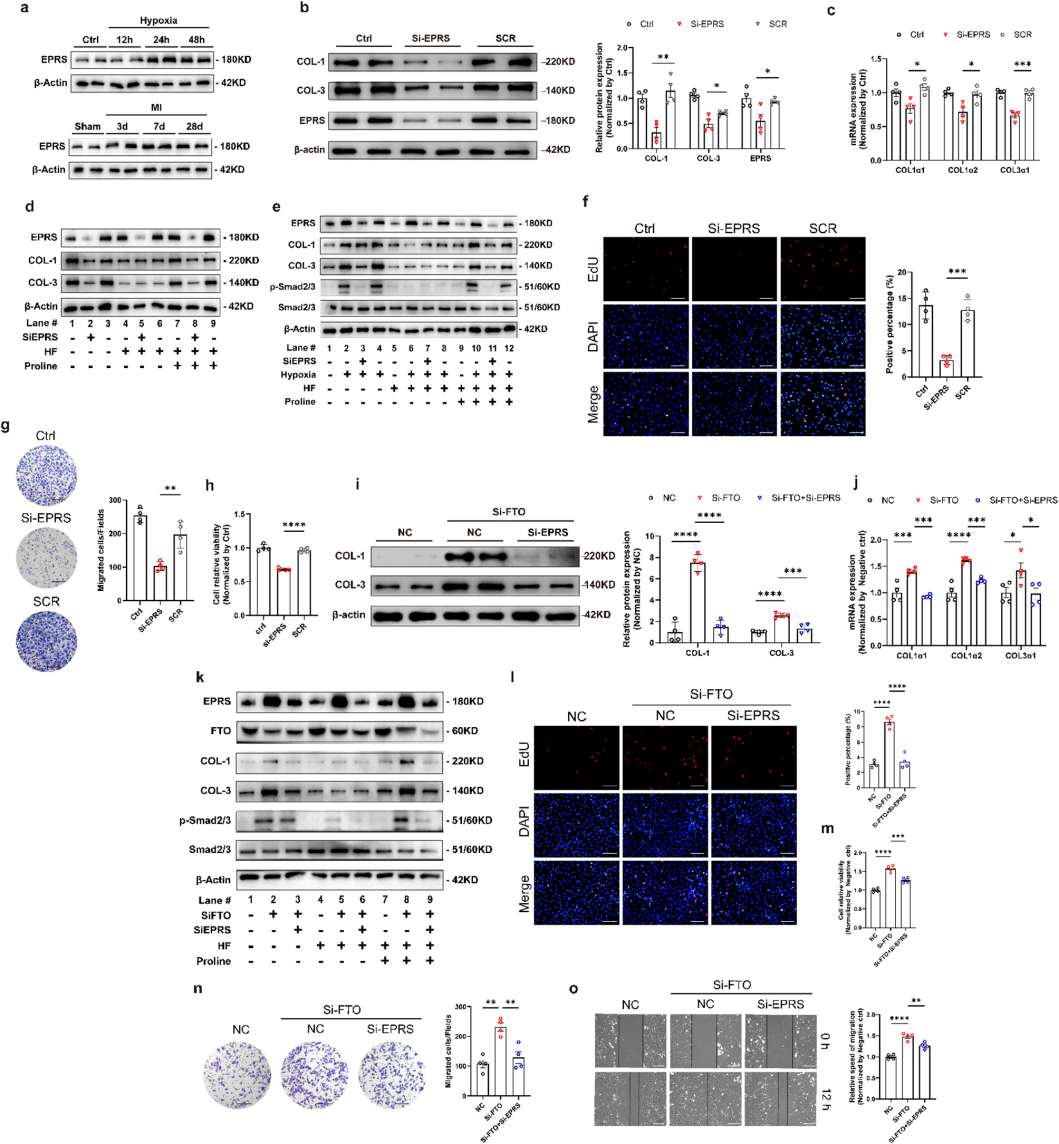
EPRS is negatively related to FTO and regulates collagen deposition in a proline-dependent manner. Expression of EPRS in a cellular model of fibrogenesis induced by hypoxia for 12, 24, and 48 h and a rat model of cardiac fibrosis 3, 7, and 28 days after MI (**a**). *n* = 4. The protein (**b**) and mRNA (**c**) levels of Collagen 1 (COL-1), Collagen 3 (COL-3), and EPRS after transfection of EPRS silencing. *n* = 4. **d**,** e** Si-EPRS cardiac fibroblasts were treated with/without hypoxia, HF (100 nM), and/or proline (2 mM) for 24 h before preparation of whole-cell extracts for immunoblotting. **f** The EdU fluorescence dying assay after transfection of Si-EPRS. Scale bar 50 μm. *n* = 4. **g** Representative images of transwell assay captured at 12 h after transfection of Si-EPRS. Scale bar 100 μm. *n* = 4. **h** Cell viability after transfection of Si-EPRS. *n* = 4. The western blot (**i**) and qRT-PCR (**j**) results of COL-1 and COL-3 after transfection with Si-FTO or co-infection with Si-FTO and Si-EPRS. *n* = 4. **k** Cardiac fibroblasts were transfected with Si-FTO or co-infection with Si-FTO and Si-EPRS, accompanied by HF (100 nM), and/or proline (2 mM) for 24 h, followed by whole-cell extracts for immunoblotting. The CCK-8 assay (**m**) and EdU fluorescence dying assay (**l**) showing the proliferation ability after transfection of Si-FTO or co-infection with Si-FTO and SiEPRS. Scale bar 50 μm. *n* = 4. The representative images of transwell assay (**n**) at 8 h and wound healing assay (**o**) at 0 and 12 h indicating the migration ability of CFs induced by NC, Si-FTO, and Si-FTO with Si-EPRS. Scale bar 100 μm. *n* = 4. SCR, scramble sequences group. NC, the negative control. The data was expressed as mean ± SEM. ^*^P < 0.05 vs. NC/SCR, ^**^P < 0.01 vs. NC/SCR, ^***^P < 0.001 vs. NC/SCR, ^****^P < 0.0001 vs. NC/SCR

Based on the above data, we found that knockdown or overexpression of FTO upregulated or downregulated the expression of EPRS at mRNA and protein levels (Fig. 5k-m). To explore the role of EPRS in regulating FTO’s function, we verified the effects of EPRS on CFs. The expression of EPRS increased 24 h after hypoxia and 7 days post-MI surgery respectively (Fig. 7a). The specific siRNA for EPRS was constructed, and the transfection efficiency was validated (Fig. 7b). Knockdown of EPRS significantly reduced the mRNA and protein expression of collagen in CFs (Fig. 7b, c). Recent studies have indicated that EPRS promoted the translation of proline-rich mRNAs via enhanced translation elongation (Wu et al. 2020). Thus, HF, an inhibitor of prolyl-tRNA synthetase and collagen I synthesis, was used to explore how EPRS affected fibrosis. The results demonstrated that EPRS-dependent collagen production could be abolished by HF therapy. Furthermore, proline supplementation partially restored collagen biosynthesis, indicating that EPRS may accelerate collagen deposition through modulating proline and prolyl-tRNA synthetase activity (Fig. 7d, e). However, the protein levels of hypoxia-mediated collagen after HF and proline treatment were lower than those with hypoxia treatment alone (Fig. 7e, lanes 2, 3, 4, 10, 11, and 12). These results implied that EPRS’s promotion of collagen deposition is partially attributable to its prolyl-tRNA synthetase activity.

The TGF-β-SMAD2/3 pathway is the essential signaling pathway in cardiac fibrosis (Derynck and Zhang 2003). Western blot was used to verify whether EPRS affected fibrosis through the TGF-β-SMAD2/3 pathway. The results showed that hypoxia treatment promoted SMAD2/3 phosphorylation, which was alleviated by EPRS knockdown (Fig. 2b, lanes 1, 2, 3, 4). In addition to collagen I and III, phosphorylated SMAD2/3 was abolished by HF with or without hypoxia treatment (Fig. 2b, lanes 5, 6, 7, 8). Moreover, additional proline partially blocked the effects of HF therapy on cardiac fibroblasts (Fig. 7e, lanes 9, 10, 11, 12). These findings indicated that EPRS modulated fibrosis through the TGF-β-SMAD2/3 pathway. In addition, EPRS silencing suppressed the proliferation of CFs using EdU fluorescence staining (Fig. 7f) and CCK-8 assays (Fig. 7h). Moreover, the migration speed of CFs decreased through Transwell assays (Fig. 7g). In all, these findings indicated that EPRS played a significant role in collagen production and the activation of CFs in a proline-dependent manner.

Subsequently, to investigate the key role of EPRS as the downstream target of FTO in cardiac fibrosis, rescue experiments were conducted to detect whether EPRS silencing could reverse the effects of FTO silencing both with and without HF and proline treatment. The results showed that EPRS knockdown markedly reduced the synthesis of collagen induced by FTO knockdown at mRNA and protein levels (Fig. 7i, j). HF treatment abolished collagen production mediated by FTO silencing, regardless of EPRS knockdown. In contrast, proline supplementation restored collagen synthesis (Fig. 7k). Moreover, the high proliferation levels mediated by SiFTO were rescued by SiEPRS (Fig. 7l, m). Knockdown of EPRS also rescued the abnormal migration speed of CFs caused by SiFTO (Fig. 7n, o). These data indicated that EPRS was required for SiFTO-induced fibrosis via proline-dependent manners.

## Discussion

m^6^A modification represents a novel form of epigenetic regulation involved in multiple cellular processes. Previous studies have reported dysregulation of m^6^A modification as a vital pathological mechanism in many cardiac diseases, mainly focusing on abnormal cardiomyocytes (Cui et al. 2023; Wang et al. 2023a, b). However, the potential involvement of m^6^A modification in CFs has not been explored. In this study, we presented the first solid evidence from both in vitro and in vivo experiments revealing the crucial role of FTO in cardiac fibrosis after MI. MI was often accompanied by hypoxia. Hypoxia-activated HIF1α bound to the FTO promoter via HRE and inhibited its expression. The downregulated expression of FTO after MI led to the abnormal activation of CFs. Forced expression of FTO suppressed CF proliferation and facilitated the deposition of collagen. Moreover, FTO overexpression alleviated cardiac fibrosis and improved cardiac function in MI rats. Mechanically, FTO demethylated m^6^A modification of fibrosis-associated transcripts including EPRS, Col1α1, and others, leading to decreased protein expression. Our findings showed that FTO may be a new target for treating cardiac fibrosis.

Previous studies have reported that the HIF1 signal pathway was enriched following MI (Janbandhu et al. 2022; Sun et al. 2020). HIF1α was a transcriptionally active protein with wide target genes related to cell repair and proliferation, hypoxia adaptation, and inflammation, all of which were vital for the function and survival of cardiac cells. Therefore, the biological effects on the enrichment of HIF1α were complex. Related studies reported that HIF1α inhibitor reduced the infarction area and improved the cardiac function in MI mice (Bao et al. 2010). However, other research demonstrated that overexpression of HIF1α in cardiomyocytes promoted vascular endothelial growth factor (VEGF) expression and accelerated angiogenesis (Datta Chaudhuri et al. 2021; Sun et al. 2018). In the present study, we found that hypoxia-induced HIF1α recruited and enriched the pathways including DNA replication, and TGF-β signaling pathway (Fig. 2i). HIF1α and HIF2α activation occurred during MI or hypoxia with reduced FTO level (Fig. 2a, b). Then, the silencing of HIF1α, instead of HIF2α, reversed the down-regulated expression of FTO under hypoxia, indicating the negative correlation between HIF1α and FTO (Fig. 2f, g). As expected, CUT&Tag-seq showed the enriched binding peaks of HIF1α in the FTO promoter (Fig. 2j). The following sequence analysis detected two potential binding sites via HRE. Subsequently, luciferase reporter assays revealed that HIF1α could recognize both sites and inhibit gene *FTO* transcription, of which Site 2 had prime effects on *FTO* transcription suppression (Fig. 2k). These findings in the present study declared that elevated HIF1α in MI might accelerate the m^6^A levels, promote CFs activation, and collagen deposition.

It is well known that overactivation of CFs and excessive collagen accumulation are key factors in the development of cardiac fibrosis. Gain- and loss-of-function experiments suggested that FTO significantly reduced collagen biosynthesis in CFs (Fig. 3). The GSEA analysis showed the negative enrichment related to collagen deposition in the FTO overexpression group (Fig. 5b). m^6^A-seq was performed to investigate the biological mechanisms of FTO in CF activation. The biological process of GO analysis showed that FTO negatively regulated DNA replication and cell migration, consistent with the results in RNA-seq (Fig. 5f). The combined results of RNA-seq and m^6^A-seq suggested that FTO might reduce collagen deposition in an m^6^A-dependent manner. Therefore, both m^6^A-seq and RNA-seq were combined to detect the specific mechanisms of FTO, suggesting EPRS may be the key point of m^6^A modification (Fig. 5j). Most studies reported that the sites of m^6^A modification were in the non-coding sequence of mRNA such as 3’-UTR (Li et al. 2023a, b, c; Huang et al. 2023). Interestingly, our finding showed that the m^6^A-modified site of EPRS was located within the coding sequence. In addition, we also found that FTO could not regulate the expression levels of all mRNAs, despite multiple m^6^A peaks within various mRNAs, suggesting selective binding of FTO to different RNAs. Therefore, future research may provide more details on the selectivity of FTO in regulating cardiac fibrosis.

EPRS is an integrated point of various pathologic targets in cardiac fibrosis. A recent study has revealed that EPRS promotes the translation of proline-rich mRNAs via enhanced translation elongation (Wu et al. 2020). EPRS was reported to regulate IL11-dependent pro-fibrotic protein translation via the ribosome stalling mechanism (Song et al. 2019; Widjaja et al. 2021). The findings showed that EPRS was closely associated with fibrosis. Knockdown of EPRS inhibited collagen accumulation and the proliferation and migration of CFs, which was consistent with the previous studies. Our finding demonstrated that FTO negatively regulated EPRS (Fig. 5k-m). EPRS silencing reversed the promotional effects induced by the silencing of FTO in CFs (Fig. 7). The findings of this study were summarized in the schematic model shown in the figure.

Our results suggested that EPRS was negatively regulated by FTO through demethylation of the m^6^A site. Previous studies detected that RNA with m^6^A sites was recognized by “readers” including YTHDF1-3, YTHDC1-2, and IGF2BP1-3 (Huang et al. 2018). m^6^A modification regulated the whole process of mRNA, including splicing, stability, degradation, and translation (Wang et al. 2022). Different m^6^A readers have different biological effects. For example, YTHDFs recognized the m^6^A-modified mRNAs and then promoted RNA degradation (Sikorski et al. 2023). On the contrary, IGF2BPs stabilized the m^6^A-modified transcripts and increased their expression (Duan et al. 2024). In this study, the decreased m^6^A modification by FTO significantly reduced the expression and half-life of EPRS mRNA, suggesting that IGF2BPs may mediate the m^6^A regulation of EPRS. Thus, we conducted the knockdowns of IGF2BP1-3. Only IGF2BP3 silencing but not others downregulated the level of EPRS (Fig. 6a; Fig. S5). Our results detected that the knockdown of IGF2BP3 could significantly reduce the half-life of EPRS mRNA degradation (Fig. 6). Furthermore, luciferase reporter assay and RIP assay confirmed that IGF2BP3 bound to EPRS mRNA. These findings demonstrated that EPRS was regulated by m^6^A modification via FTO and IGF2BP3.

Surprisingly, IGF2BP3 silencing did not affect the protein levels of COL-1 and COL-3 induced by Si-FTO (Fig. 6b, c), indicating that COL-1 and COL-3 might not be the direct targets of IGF2BP3. IGF2BP3 was an m6A “reader” protein that could bind to numerous specific m^6^A sites, leading to the degradation of target mRNA (Li et al. 2023a, b, c; Zhang et al. 2022). IGF2BP3 may recognize and bind to other m^6^A sites on the mRNA of collagen-related genes during MI. A recent study reported that IGF2BP3-mediated post-transcriptional regulation is correlated with cardiomyocyte regeneration, suggesting protective effects of IGF2BP3 after MI (Li et al. 2023a, b, c). This may explain why IGF2BP3 stabilized EPRS mRNA level but did not affect collagen biosynthesis.

Several limitations should be focused on. First, we only detected the elevated HIF signal pathway in MI/hypoxia, and it remained unknown whether other transcription factors co-regulated FTO transcription. Second, the present study has confirmed that FTO decreased EPRS mRNA in an IGF2BP3-dependent manner. Nevertheless, the findings could not exclude the possibility that FTO mediated a decrease in EPRS through other post-transcriptional regulation, including the inhibition of mRNA translation. The latest research has suggested that m^6^A recognition protein containing IGF2BP3 promoted the translation efficiency of target mRNA (Shan et al. 2023; Tang et al. 2023). Thus, we could not exclude the possibility of other involved molecular mechanisms. Third, although the above experiments in vitro indicated that EPRS silencing inhibited collagen deposition and rescued the FTO-mediated CFs activation, it remained unclear whether EPRS inefficiency could ameliorate cardiac fibrosis during MI. Collectively, this study made it clear that FTO suppresses cardiac fibrosis after myocardial infarction via m^6^A-mediated epigenetic modification of EPRS.

## Conclusion

We uncovered the link between cardiac fibrosis and m^6^A modification induced by FTO for the first time. HIF1α activated by MI/hypoxia bound to the FTO promoter and reduced its expression level, leading to the aberrant m^6^A modification. EPRS, negatively affected by FTO via IGF2BP3-mediated m^6^A modification, was critical in regulating cardiac fibrosis after MI. The findings implied the epigenetic mechanisms of cardiac fibrosis and provided new insight into therapeutic strategies for cardiac fibrosis.

## Electronic supplementary material

Below is the link to the electronic supplementary material.

Supplementary Material 1

Supplementary Material 2

## Data Availability

The datasets supporting the conclusions of this article are available in the NCBI Gene Expression Omnibus (https://www.ncbi.nlm.nih.gov/geo/) and are accessible through the GEO Series accession number GSE259328 and GSE257546.

## Abbreviations

CFs: Cardiac fibroblasts
COL-1: Collagen I
COL-3: Collagen III
EPRS: Glutamyl-prolyl-tRNA synthetase
HF: Halofuginone
m^6^A: *N*^6^-methyladenosine
METTL14: Methyltransferase like 14
METTL3: Methyltransferase like 3
METTL16: Methyltransferase like 16
FTO: Fat body mass and obesity-associated protein
ALKBH5: A-ketoglutarate-dependent dioxygenase homology 5
YTHDF: YT521-B homology domain-containing family proteins
YTHDC: YTH domain-containing proteins
IGF2BP: insulin-like growth factor 2 binding proteins
MI: Myocardial infarction
NRCFs: Neonatal rat cardiac fibroblasts
DMEM: Dulbecco’s Modified Eagle Medium
LAD: Left anterior descending coronary artery
RNA-seq: Transcriptome sequencing
m^6^A-seq: Methylated RNA immunoprecipitation sequencing
SEM: standard error
FBN: Fibronectin
α-SMA: α-smooth muscle actin
qRT-PCR: Quantitative real-time PCR
TSS: Transcriptional start site
HRE: Hypoxia transcriptional response elements
EF: Ejection fraction
FS: Fractional shortening
GSEA: Gene set enrichment analysis
GO: Gene Ontology
CDS: Coding region
KEGG: Kyoto Encyclopedia of Genes and Genomes
IGV: Integrative Genomics Viewer
VEGF: Vascular endothelial growth factor
