# Supplementary material for: FTO suppresses cardiac fibrosis after myocardial infarction via m6A-mediated epigenetic modification of EPRS
Source: Mol Med. 2024 Nov 13;30:213. doi: 10.1186/s10020-024-00985-7 (PMC11562098; doi:10.1186/s10020-024-00985-7)
Supplement: Supplementary file 1 — Supplementary Material 1 [file 10020_2024_985_MOESM1_ESM.docx]

**Supplementary Materials**

FTO suppresses cardiac fibrosis after myocardial infarction via m^6^A-mediated epigenetic modification of EPRS

Jian Wang^1^, Yanyan Li^2^, Lijie Deng^1^, Yafang Zha^1^, Song Zhang^1*^

^1^Department of Cardiology, Renji Hospital Affiliated Shanghai Jiao Tong University School of Medicine, Shanghai, 200127, People’s Republic of China.

^2^Department of Cardiology, Xinhua Hospital Affiliated Shanghai Jiao Tong University School of Medicine, Shanghai, 200092, People’s Republic of China.

*Correspondence and requests for materials should be addressed to S.Z. (email: zhangsong3961@xinhuamed.com.cn)

**Supplementary Methods**

**Plasmids, RNA knockdown, and transfection**

FTO, EPRS, HIF1α, HIF2α, IGF2BP1, IGF2BP2, and IGF2BP3 siRNAs were purchased from ZuorunTech (Shanghai, China). The most validated sequences of siRNA were used for subsequent assays. Rat FTO cDNA was amplified by PCR and cloned into the pcDNA 3.1 (Invitrogen, USA) vector according to the manufacturer’s instructions (ZuorunTech, China). NRCFs were transfected for 48 hours with siRNA (100 nM) or plasmids (1 μg/ml) performed by Hieff Trans^®^siRNA transfection reagent (Yeasen, China) or lipofectamine 3000 (Thermo Fisher Scientific, USA) respectively. The scramble sequences or the empty plasmids were transfected as a negative control.

**Quantitative real-time PCR (qRT-PCR)**

Total RNA was extracted from NRCFs and rat heart tissue using TRIzol (Takara, Japan). Total RNA was reversely transcribed to cDNA with PrimeScript™ RT Master Mix Reagent (Takara, Japan). The expression levels of mRNAs relative to ACTB were performed by the ABI 7500 Real-Time PCR system (Applied Biosystems, USA) using SYBR Green PCR Master Mix (Yeasen, China).

**Western blot**

Total protein samples collected from NRCFs and rat heart tissue were prepared by RIPA buffer (Beyotime, China) containing protease inhibitors (Beyotime, China). Proteins were separated on SDS-PAGE and transferred to the nitrocellulose membrane (Millipore, USA). After being blocked with 5 % non-fat milk for 1 hour, the membranes were incubated with the primary antibody overnight at 4 ℃. Then the membranes were washed and incubated with a secondary anti-rabbit or anti-mouse polyclonal antibody (Beyotime, China). Signals were revealed by enhanced chemiluminescence ECL (Thermo Scientific) on the image capturer (Tanon 5200) and quantified by densitometry software (Image-Pro Plus). The primary antibodies used in the study were attached in Table S1.

**Cell proliferation assay**

Cell proliferation assay was carried out by CCK-8 assay and EdU fluorescence staining. For the CCK-8 assay, NRCFs were cultured in 96-well plates at a density of 5×10^3^ cells/well. After the corresponding treatment, NRCFs were subjected to 10 μl CCK-8 solution (Beyotime, China) and incubated at 37 ℃ for 1h. The optical density was measured by the microplate reader (BioTek, USA) at 450 nm.

The new synthesized DNA in NRCFs was recorded by BeyoClick^TM^ EdU Cell Proliferation Kit (Beyotime, China) followed by the manufacturer’s instructions. NRCFs were cultured with 10 μM 5-ethynyl-2' -deoxyuridine (EdU) medium for 2 hours, fixed with 4 % paraformaldehyde (Biosharp, China), and penetrated with 0.5 % Triton X-100 (Beyotime, China). NRCFs were incubated with the staining complex and DAPI solution. The fluorescence images were acquired under the fluorescence microscope (Zeiss, Germany).

**Cell migration assay**

Cell migration assay was observed using the transwell migration and wound healing assay. NRCFs cultured with serum-free medium (4×10^4^ cells/200μl) were seeded into the upper chamber (Corning, USA), and the medium containing 10 % FBS was added into the lower chamber as the attachment. After 12 hours of culture, the upper chamber of NRCFs was removed. Cells of the lower chamber were fixed with 4 % paraformaldehyde, penetrated with methanol, and stained in crystal violet solution (Beyotime, China). The stained cells were counted under the microscope (Olympus, Japan).

For the wound healing assay, NRCFs were plated in 6-well plates after the corresponding treatment. Cells were scratched with a pipette tip (200 μl) and a ruler when reaching 90 % confluence. Subsequently, the medium was replaced by the serum-free media. Images were obtained at 0 and 24 hours after scratch.

**Echocardiographic measurements**

4 weeks after the MI surgery, transthoracic echocardiography was performed with the Vevo 2100 High-Resolution Imaging System (Visual Sonics, Canada) to measure changes in the left ventricular function. M-mode tracing of the left ventricle was recorded from the parasternal long-axis view. Ventricular parameters were recorded and analyzed including left ventricular ejection fraction (EF) and fractional shortening (FS).

**Masson’s trichrome staining**

The rat hearts were collected and immersed in 4 % paraformaldehyde overnight. Subsequently, the hearts were embedded with paraffin and cut into 5 μm thick cross-sectional slices along the center of the fibrotic scar. The slices were stained by Masson’s trichrome staining kit (ServiceBio, China). Fibrotic heart tissue was stained blue and myocardium red. The percentage of fibrotic tissue was measured by Image-Pro Plus.

**RNA immunoprecipitation**

RNA immunoprecipitation (RIP) kit (BersinBio, China) was used according to the manufacturer’s instructions. Cells were lysed with RIP lysis buffer. Subsequently, the complex of RNA and protein was immunoprecipitated by anti-IGF2BP3 antibody (Abcam, USA) and rabbit immunoglobulin G (Proteintech, China). Then, RNA was separated, purified, and reversely transcribed. The relative interaction between protein and RNA was detected by qRT-PCR and agarose gel electrophoresis.

**Luciferase reporter assay**

Sequences from EPRS containing the wild-type m^6^A motifs (EPRS-WT) and mutant m^6^A motifs (m^6^A was replaced by C, EPRS-MUT) were subcloned into pmirGLO luciferase vector (Sangon Biotech, China). NRCFs were seeded in 24-well plates and transfected with EPRS-WT or EPRS-MUT for 48 hours. Subsequently, luciferase activity was measured by a Dual-Luciferase Reporter Gene Assay Kit (Yeasen, China). Relative Firefly luciferase activity was normalized by the Renilla luciferase.

**mRNA stability assays**

Actinomycin D (5 μg/ml) (MCE, USA) was added to block mRNA transcription. Cells were harvested after incubation for 0, 3, and 6 hours. The total RNA was harvested for reverse transcription. The mRNA transcription levels were measured with qRT-PCR and the half-life of mRNA was detected.

**Supplementary Figures**


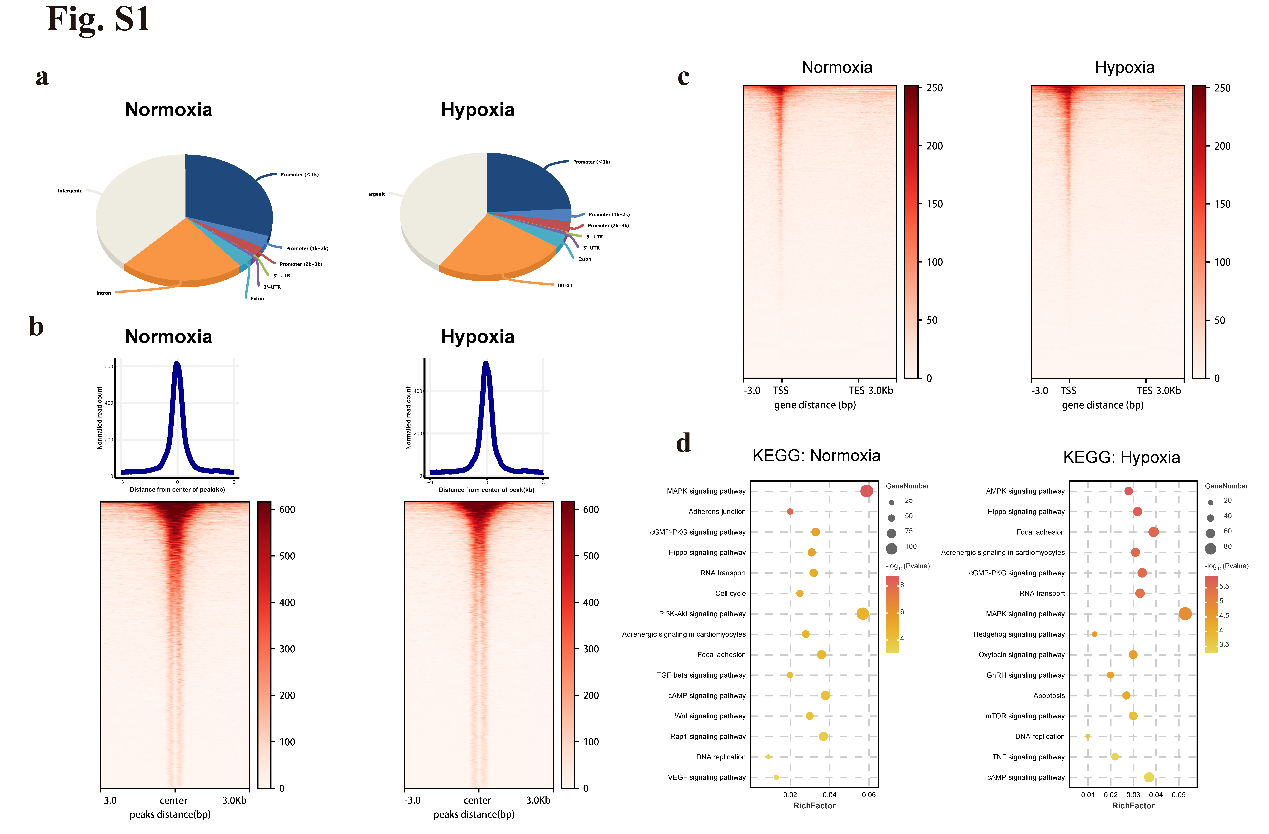


**Fig. S1** **a** and **b,** Distribution of peaks from CUT&Tag-seq reads in cardiac fibroblasts under control or hypoxia treatment. Heatmaps (**c**) and enrichment maps (**d**) of KEGG from CUT&Tag-seq reads in cardiac fibroblasts.


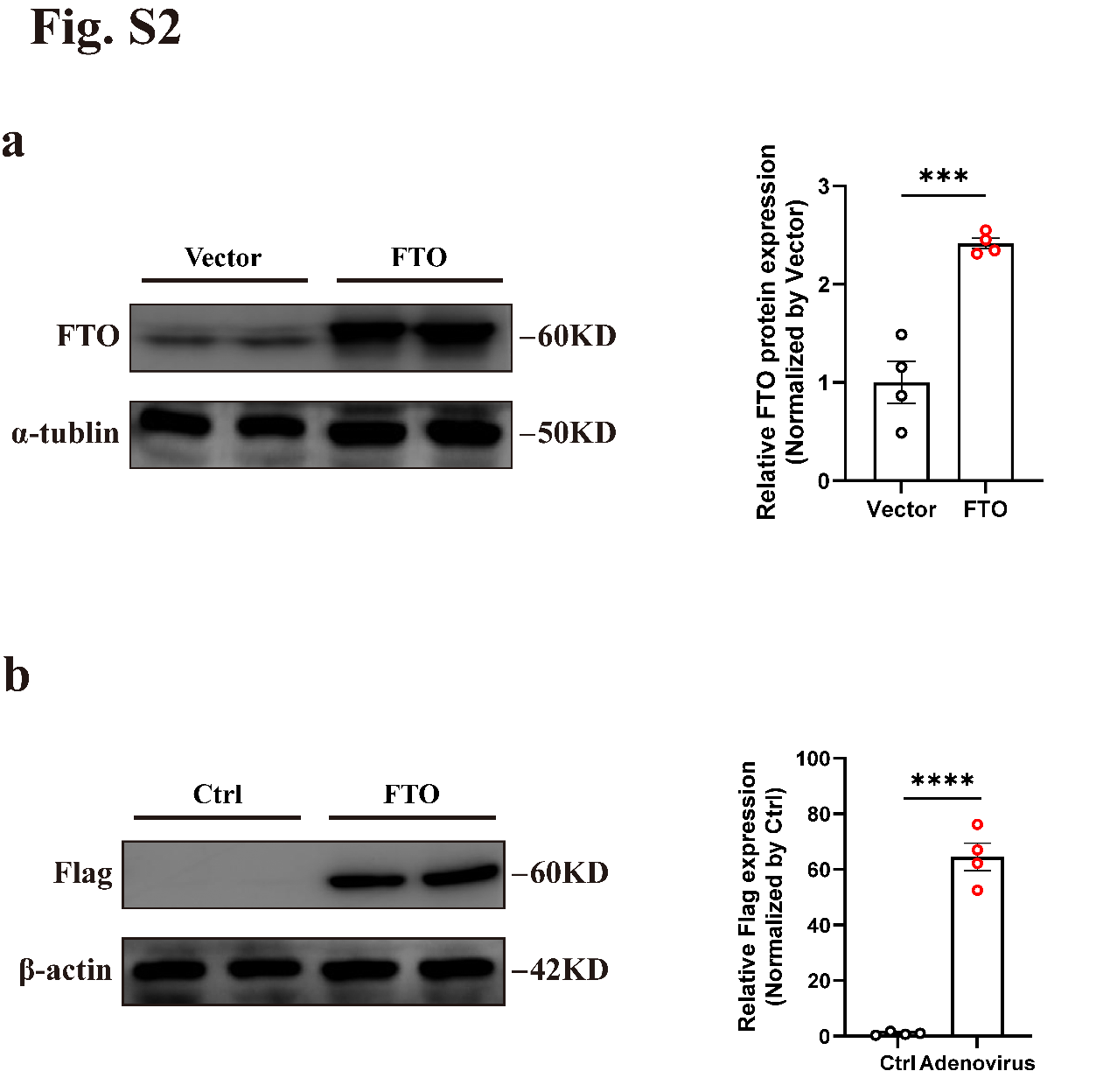


**Fig. S2** The transfection efficiency of FTO-overexpressing plasmids and adenovirus in rats. **a** The relative protein expression of FTO after transfection of FTO-overexpressing plasmids. *n*=4. **b** The relative expression of Flag protein in rats 7 days after transfection of adenovirus. Vector, the empty plasmid. The data was expressed as mean ± SEM. ^***^ *P* < 0.001 vs Vector. ^****^ *P* < 0.001 vs Ctrl.


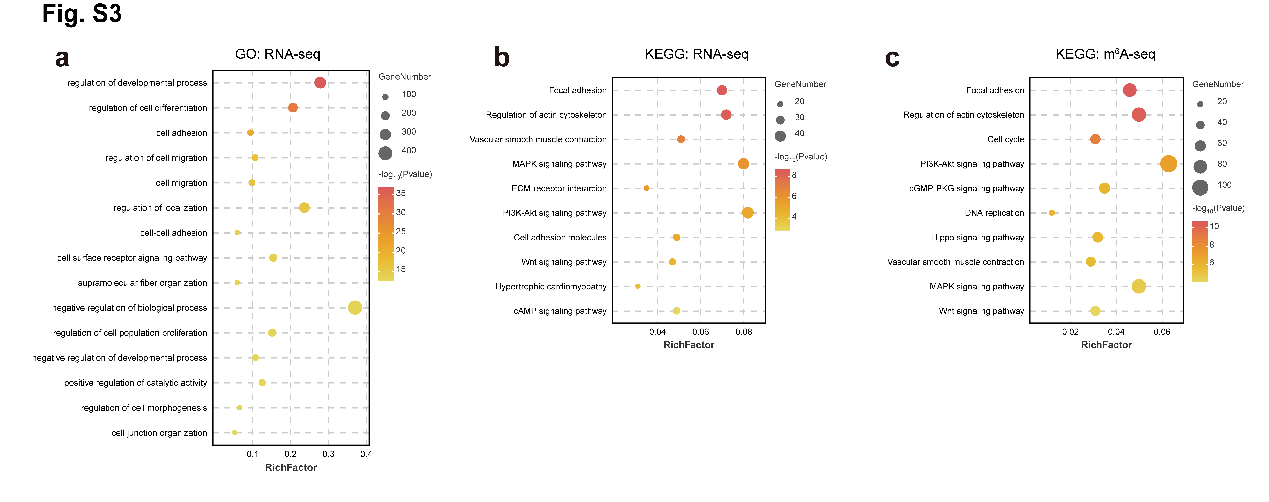


Fig. S3 **a** Down-regulated enrichment maps of biological process from RNA-seq reads in cardiac fibroblasts. The down-regulated enrichment maps of KEGG in RNA-seq (**b**) and m^6^A-seq (**c**) respectively.


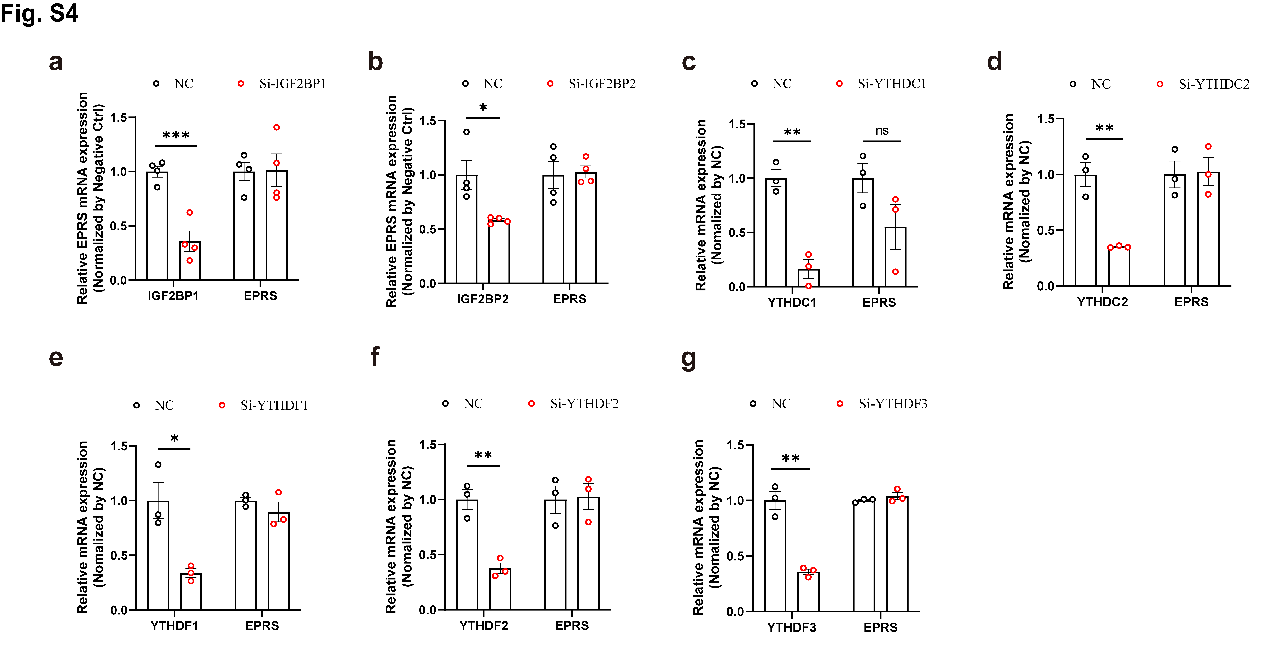


**Fig. S4** The mRNA levels of EPRS with transfection of siRNAs for IGF2BP1 (**a**), IGF2BP2 (**b**), YTHDC1 (**c**), YTHDC2 (**d**), YTHDF1 (**e**), YTHDF2 (**f**), and YTHDF3 (**g**). *n*=3. NC, the negative control. The data was expressed as mean ± SEM. ^*^ *P* < 0.05 vs NC. ^**^ *P* < 0.01 vs NC.

**Supplementary Tables**

**Table S1.** Detailed information on antibodies used in the study

| Antibodies | Dilution | Company | Catalog |
| --- | --- | --- | --- |
| FTO | 1：1000 | Abcam | ab280081 |
| Collagen I | 1：1000 | Abclonal | A1352 |
| Collagen III | 1：1000 | Abclonal | A0817 |
| β-actin | 1：2000 | Abclonal | AC004 |
| EPRS | 1：5000 | Proteintech | 67712-1-Ig |
| IGF2BP3 | 1：1000 | Abcam | ab177477 |
| HIF1α | 1：1000 | Abcam | ab179483 |
| HIF2α | 1：1000 | Proteintech | 83790-1-RR |
| GAPDH | 1：2000 | Abclonal | AC027 |
| M^6^A | 1：4000 | SySy | 202003 |
| Fibronectin | 1：50 | Proteintech | 15613-1-AP |
| α-SMA | 1：50 | Proteintech | 67735-1-Ig |
| Smad2/3 | 1:1000 | Cell Signaling Technology | 8685S |
| Phospho-SMAD2 (Ser465/467)/SMAD3 (Ser423/425) | 1:1000 | Cell Signaling Technology | 8828S |

**Table S2.** The sequences of primers used for real-time PCR

| Gene |  | Sequences | |
| --- | --- | --- | --- |
| FTO | Forward | | 5’- GCTGTGGAAGAAGATGGAGAGTGTG -3’ |
|  | Reverse | | 5’- ATCAGGACGGCAGACAGAATTTCAC -3’ |
| Col1α1 | Forward | | 5’- TGTTGGTCCTGCTGGCAAGAATG -3’ |
|  | Reverse | | 5’- GTCACCTTGTTCGCCTGTCTCAC -3’ |
| Col1α2 | Forward | | 5’- CCGAGGCAGAGATGGTGTTGATG -3’ |
|  | Reverse | | 5’- TGAGCAGCAAAGTTCCCAGTAAGAC-3’ |
| Col3α1 | Forward | | 5’- AGTCGGAGGAATGGGTGGCTATC -3’ |
|  | Reverse | | 5’- CAGGAGATCCAGGATGTCCAGAGG -3’ |
| EPRS | Forward | | 5’- GCCTGACAACTCGCACTATCGG -3’ |
|  | Reverse | | 5’- TACCACCTGGACAGACGCTACAC -3’ |
| β-actin | Forward | | 5’- TGTCACCAACTGGGACGATA -3’ |
|  | Reverse | | 5’- GGGGTGTTGAAGGTCTCAAA -3’ |
| IGF2BP3 | Forward | | 5’- ATTCCAAGTTCCATCCACTCCATTC -3’ |
|  | Reverse | | 5’- AGTTCTGGGTGTCTGTGCTCTG -3’ |
| HIF1α | Forward | | 5’- CACCGCCACCACCACTGATG -3’ |
|  | Reverse | | 5’- TGAGTACCACTGTATGCTGATGCC -3’ |
| HIF2α | Forward | | 5’- CCTGCTGTCCTGCCTCATCATC -3’ |
|  | Reverse | | 5’- GGTGAACTTCATGTCCATGCTGTG -3’ |
| METTL3 | Forward | | 5’- CTCTCGTAACCTATGCTGACCACTC -3’ |
|  | Reverse | | 5’- CCACTGTAGTCAAGTCCTGCTCTG -3’ |
| METTL14 | Forward | | 5’- CGAAGTCACCTCCTCCCAAATCC -3’ |
|  | Reverse | | 5’- CGACCACGGCCAGCAGATG -3’ |
| WTAP | Forward | | 5’- GAACATCCTTGTCATGCGGCTAG -3’ |
|  | Reverse | | 5’- CGGCTGCTGAACTTGCTTGAG -3’ |
| ALKBH5 | Forward | | 5’- GGGACCACCAAACGGAAGTACC -3’ |
|  | Reverse | | 5’- CTCCTCCTCCTTCTGCAACTGATG -3’ |

**Table S3.** The sequences of primers used for CUT&Tag-qPCR

| CUT&Tag site |  | | Sequences | |
| --- | --- | --- | --- | --- |
| Site 1 | | Forward | | 5’- AGTCATATATGTGTGCATGTGC -3’ |
|  |  | Reverse | | 5’- AGCCTGCACTACACACATAAAC -3’ |
| Site 2 | | Forward | | 5’- AAGTGGTCCCTTGTGCTCTG -3’ |
|  |  | Reverse | | 5’- TGAGACTTCTGAAACCAAGAGC -3’ |
