# Supplementary material for: FTO suppresses cardiac fibrosis after myocardial infarction via m6A-mediated epigenetic modification of EPRS
Source: Mol Med. 2024 Nov 13;30:213. doi: 10.1186/s10020-024-00985-7 (PMC11562098; doi:10.1186/s10020-024-00985-7)
Supplement: Supplementary file 2 — Supplementary Material 2 [file 10020_2024_985_MOESM2_ESM.pdf]

Figure 1 a

m^{6}A dot blot

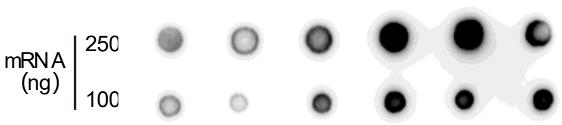

Methylene blue

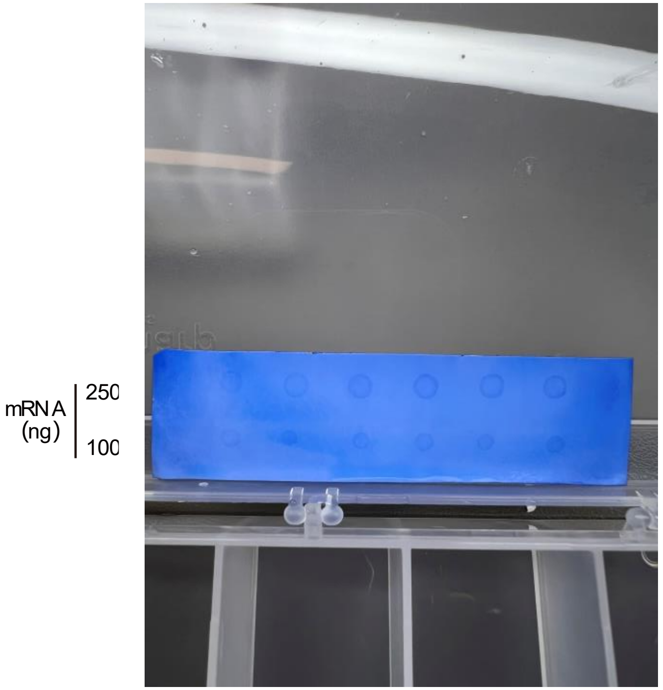

Figure 1 b

m^{6}A dot blot

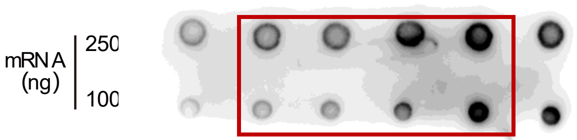

Methylene blue

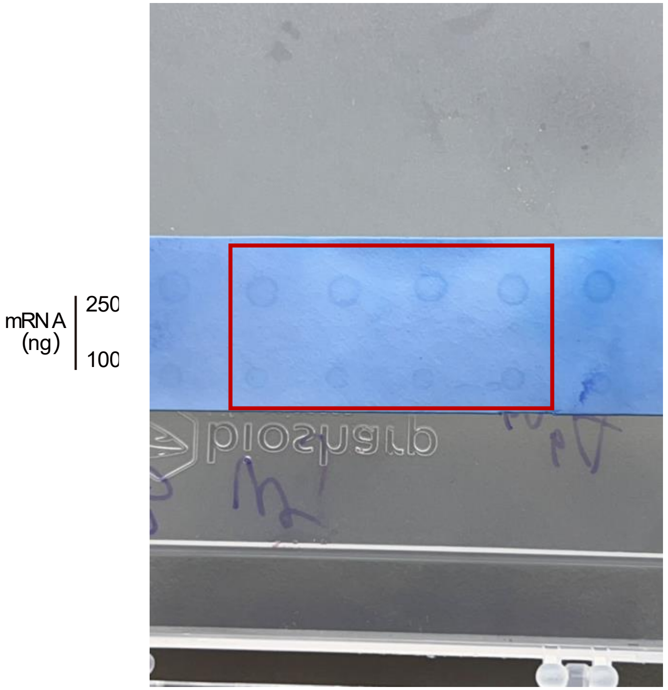

Figure 1 h

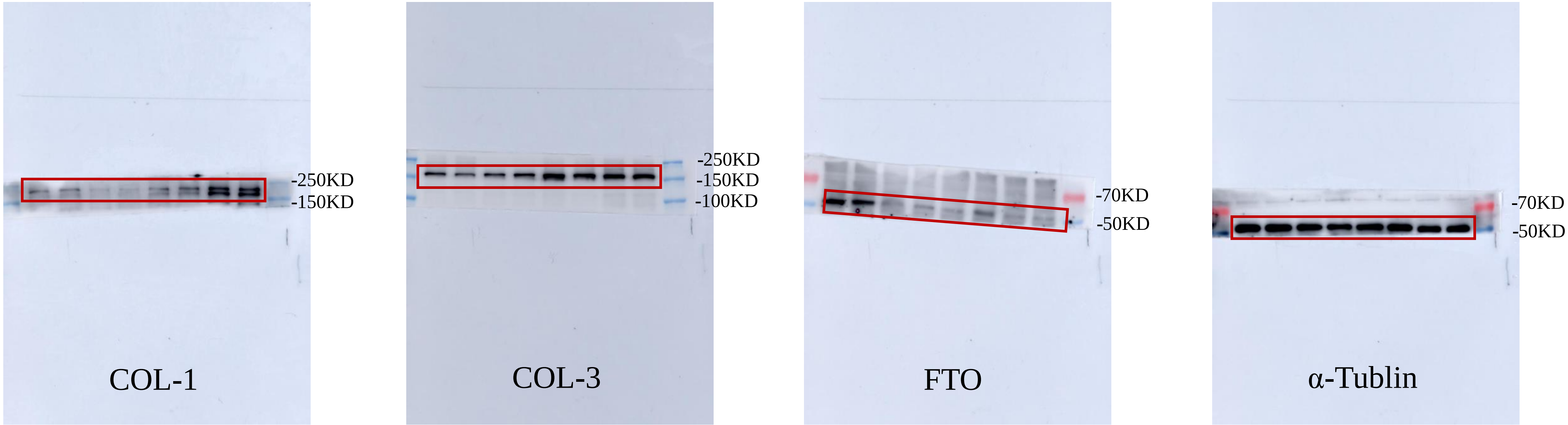

-250KD
-150KD
COL-1
-250KD
-150KD
-100KD
COL-3
-70KD
-50KD
FTO
-70KD
-50KD
α-Tublin

Figure 1 j

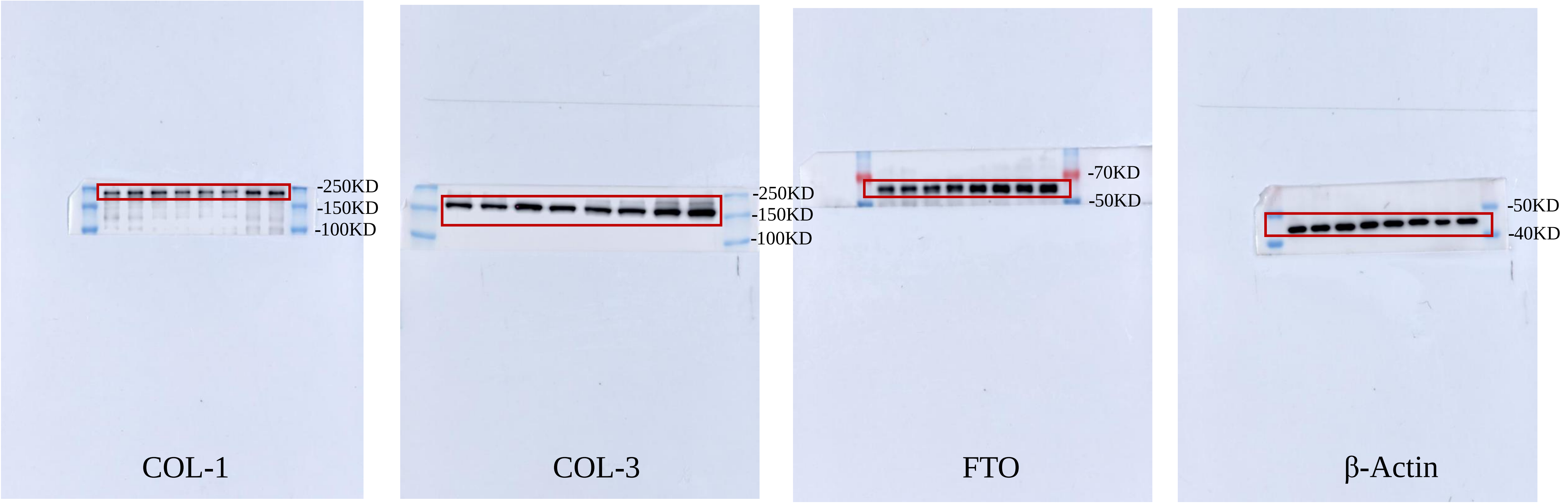
-250KD
-150KD
-100KD
COL-1
-250KD
-150KD
-100KD
COL-3
-70KD
-50KD
FTO
-50KD
-40KD
β-Actin

Figure 2 a

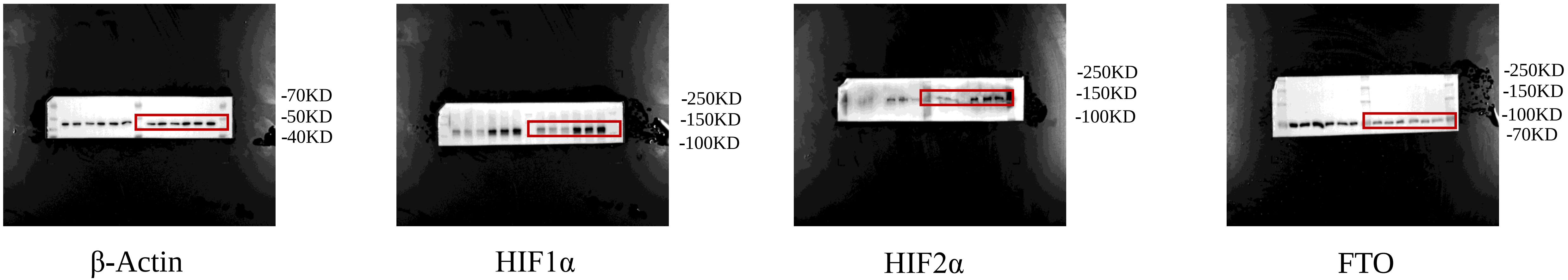

Figure 2 c

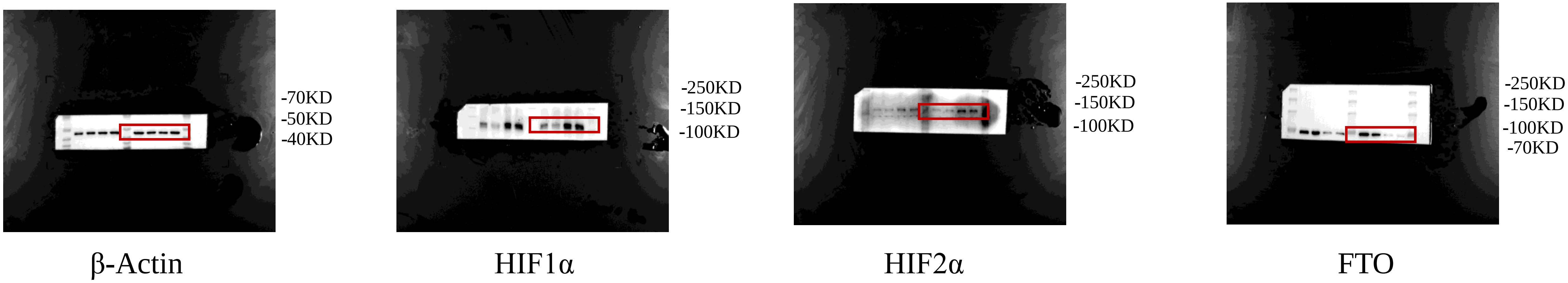

Figure 2 g Si-HIF1α

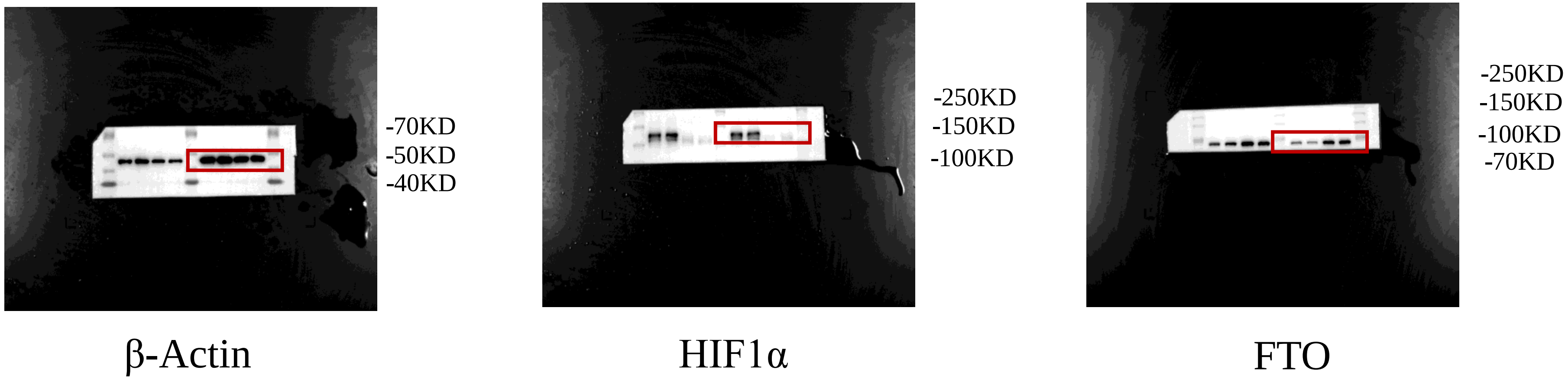

Figure 2 g Si-HIF2α

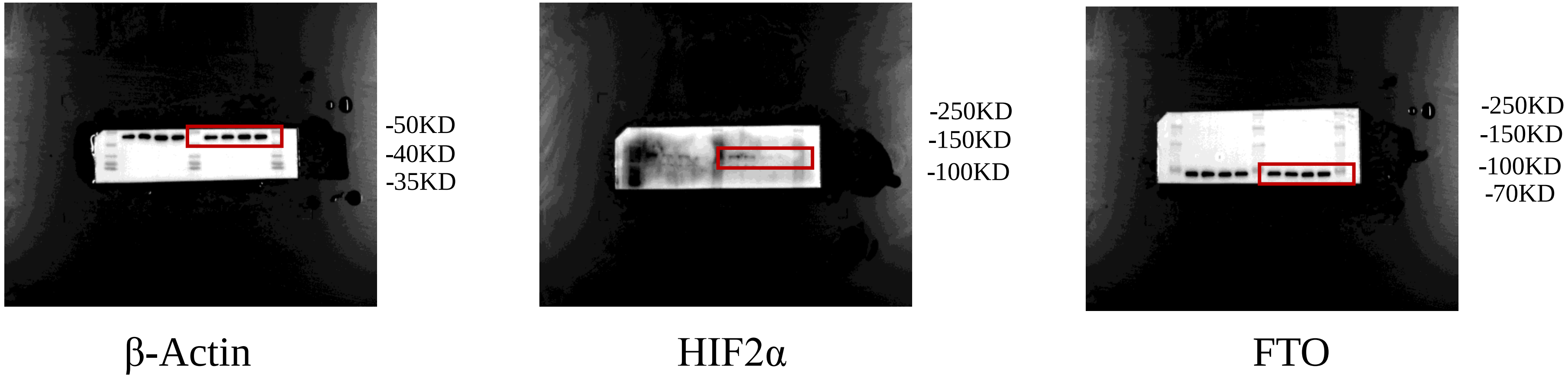

Figure 3 b

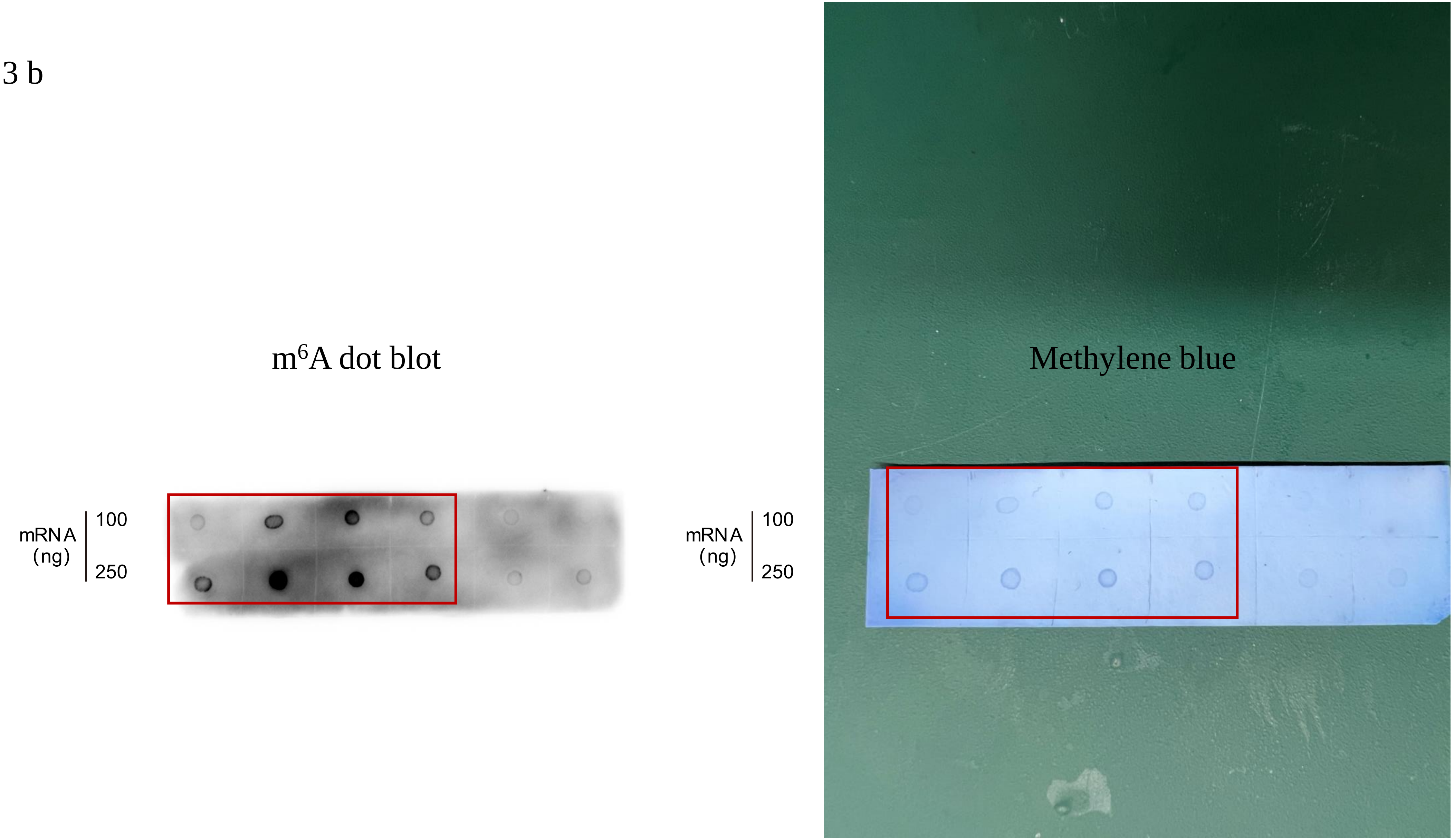

Figure 3 c

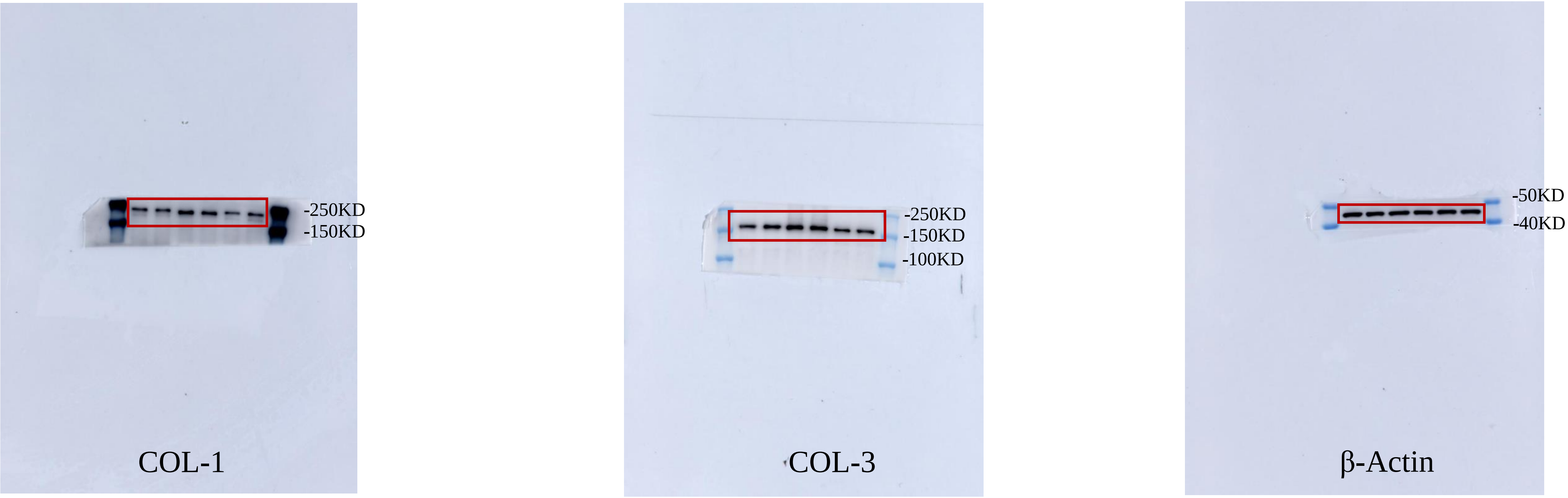

Figure 3 e

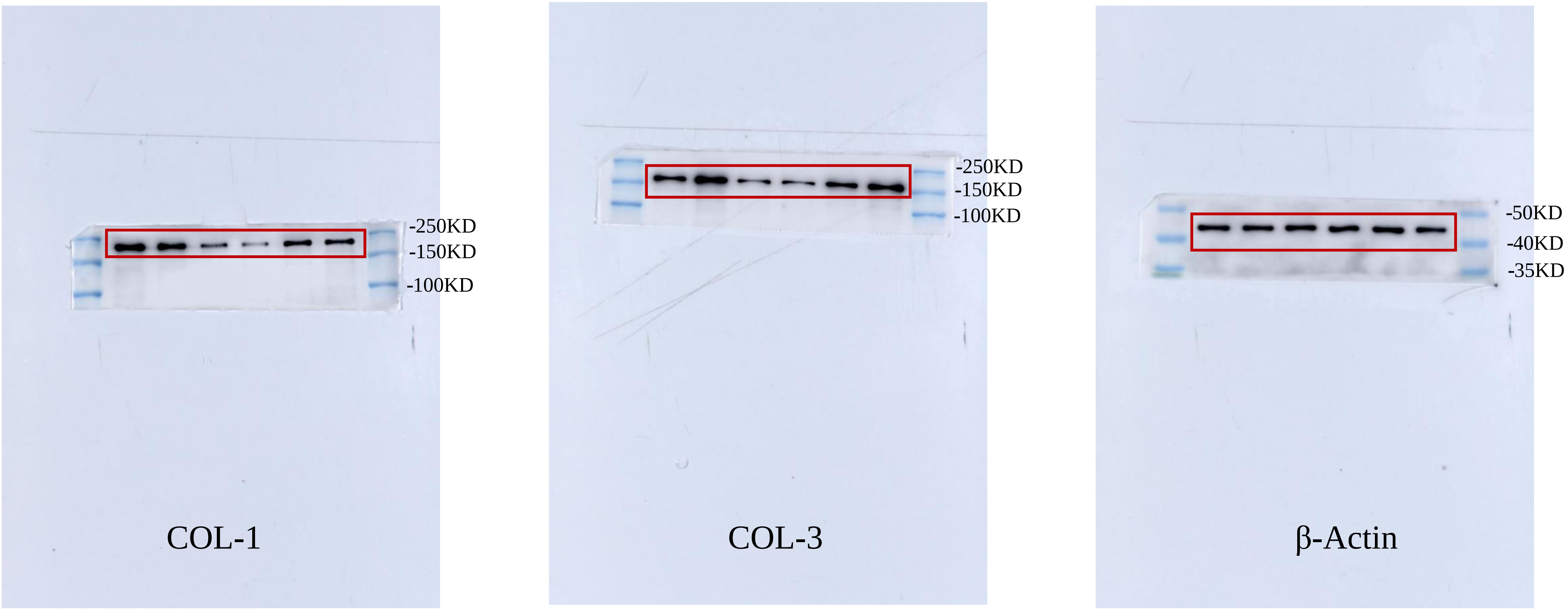
-250KD
-150KD
-100KD
COL-1
-250KD
-150KD
-100KD
COL-3
-50KD
-40KD
-35KD
β-Actin

Figure 4 a

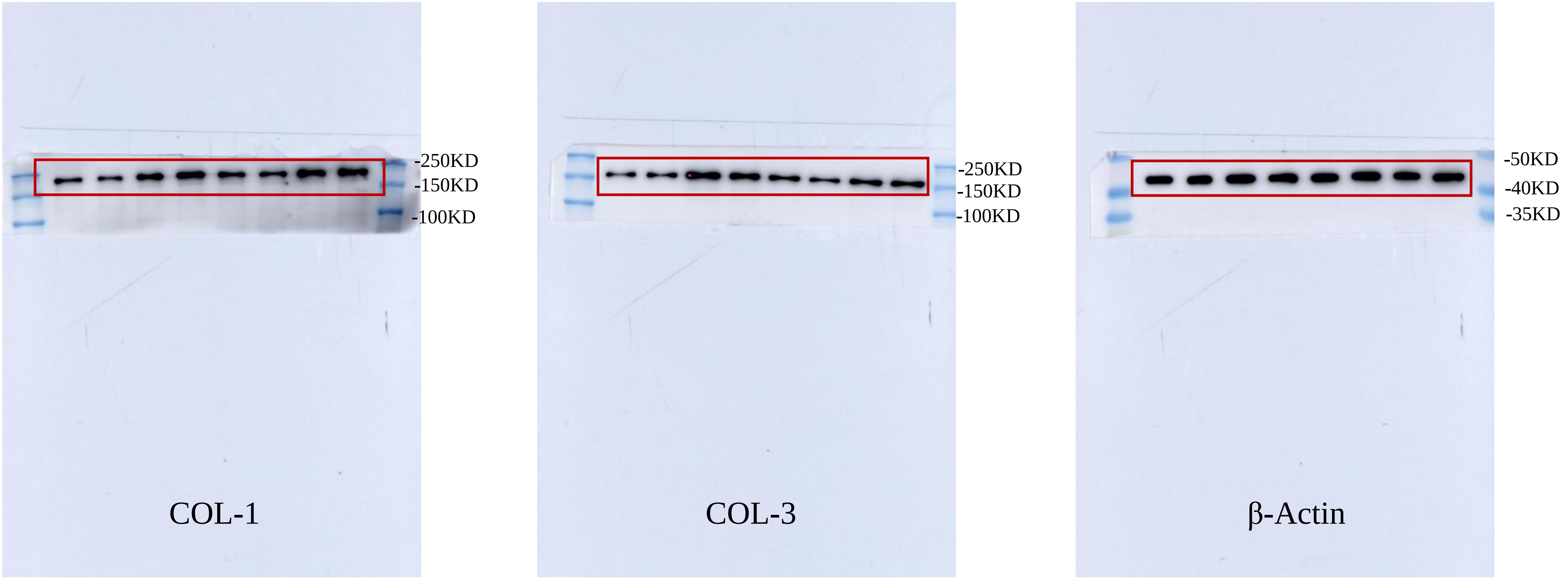
-250KD
-150KD
-100KD
COL-1
-250KD
-150KD
-100KD
COL-3
-50KD
-40KD
-35KD
β-Actin

Figure 4 g

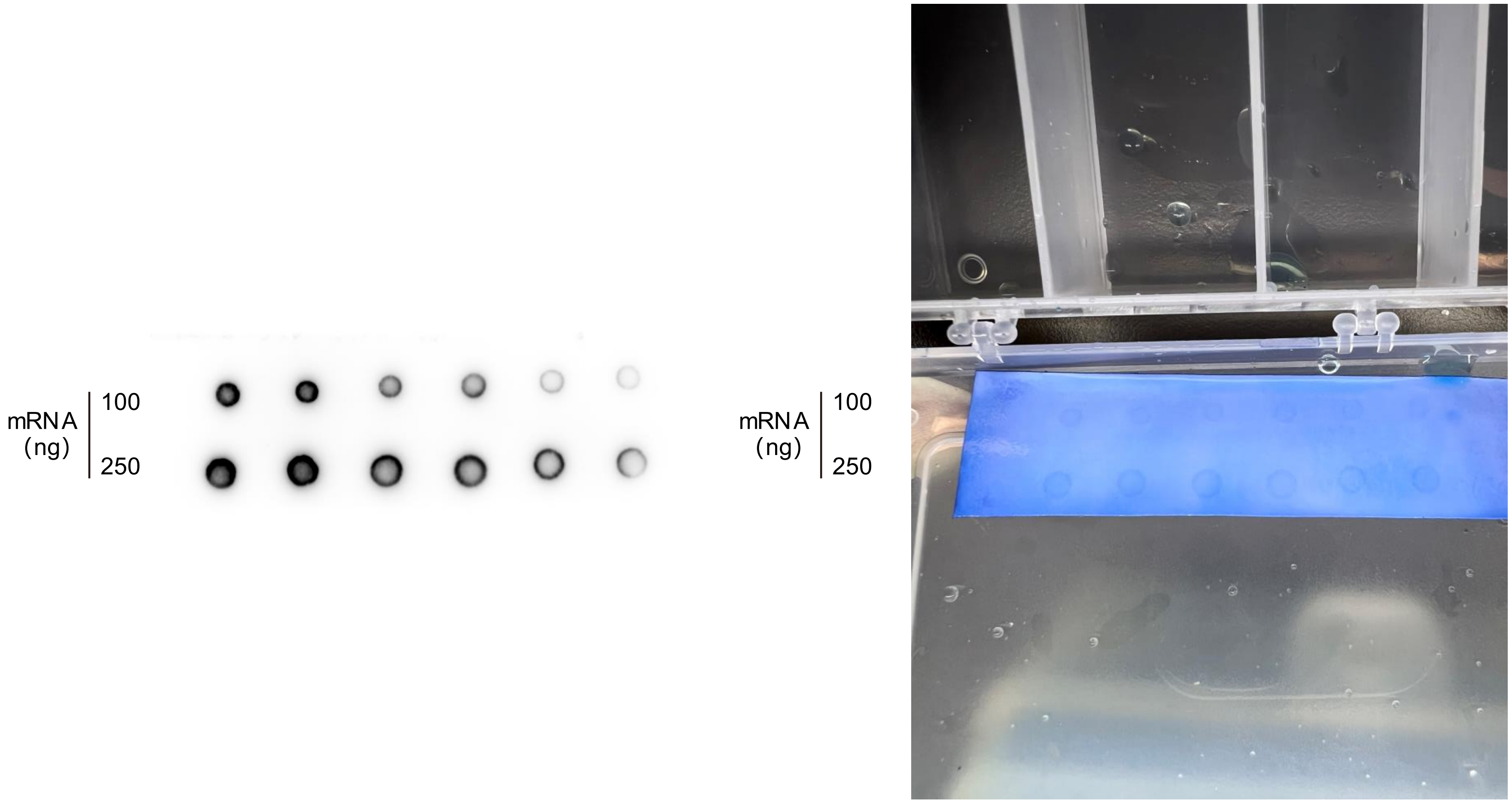

Figure 4 j

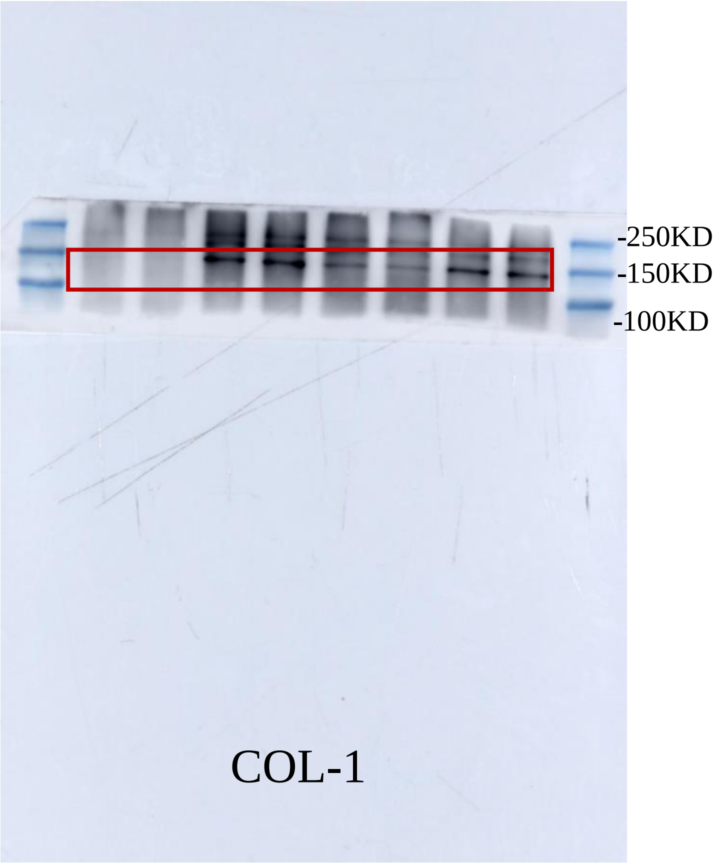
-250KD
-150KD
-100KD
COL-1

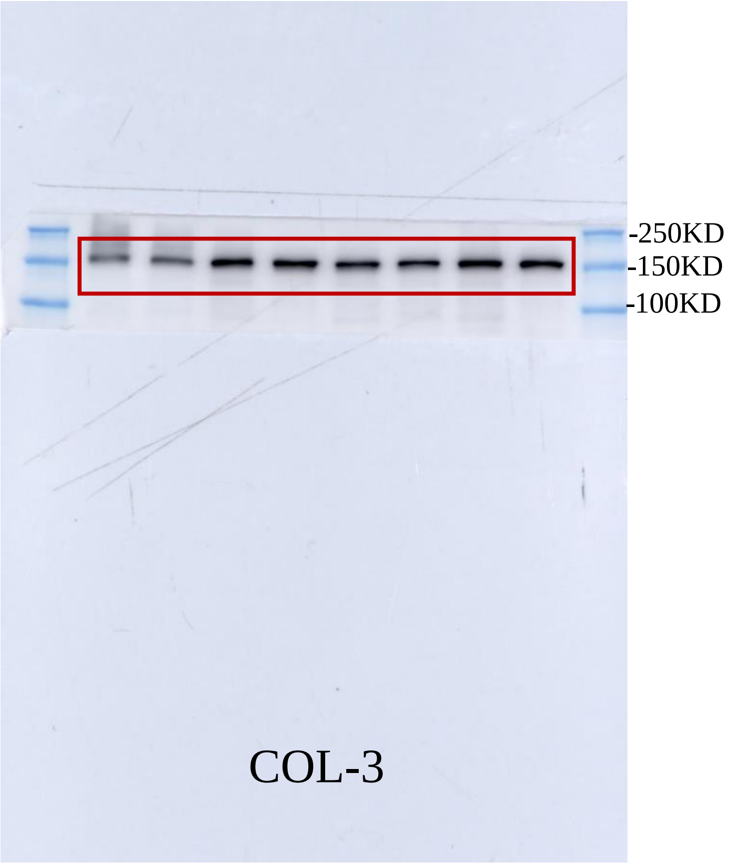
-250KD
-150KD
-100KD
COL-3

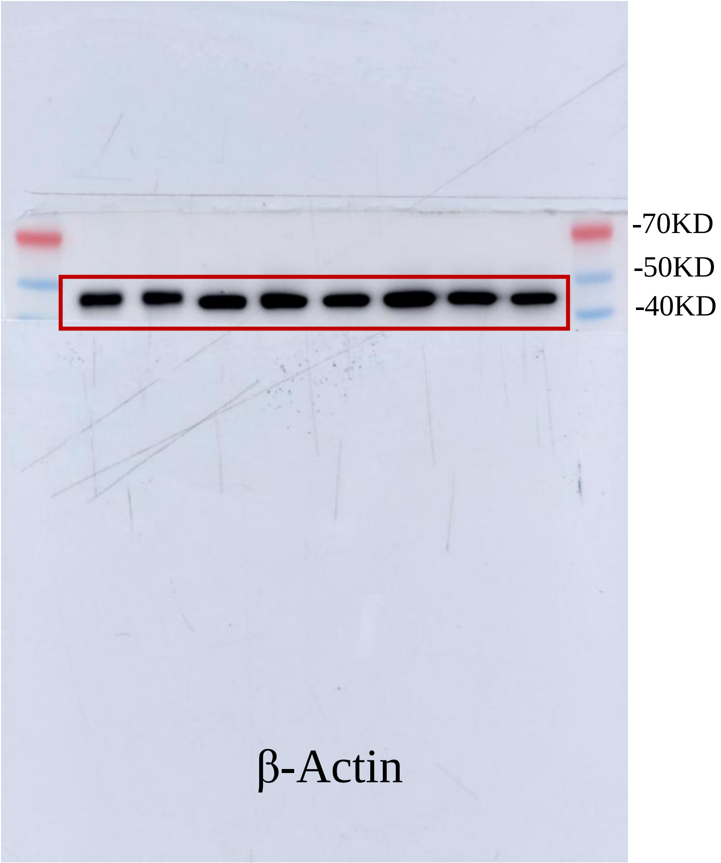
-70KD
-50KD
-40KD
β-Actin

Figure 4 k

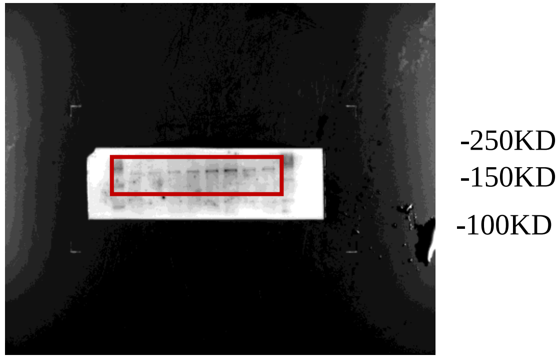

COL-1

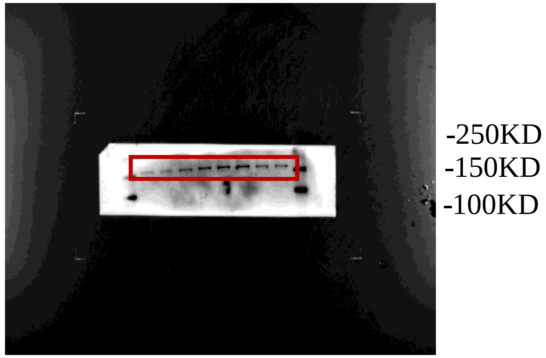

COL-3

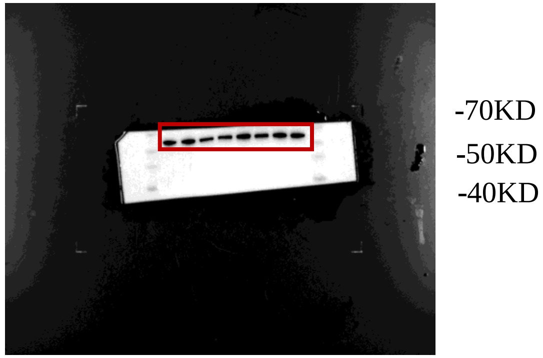

β-Actin

Figure 5 c

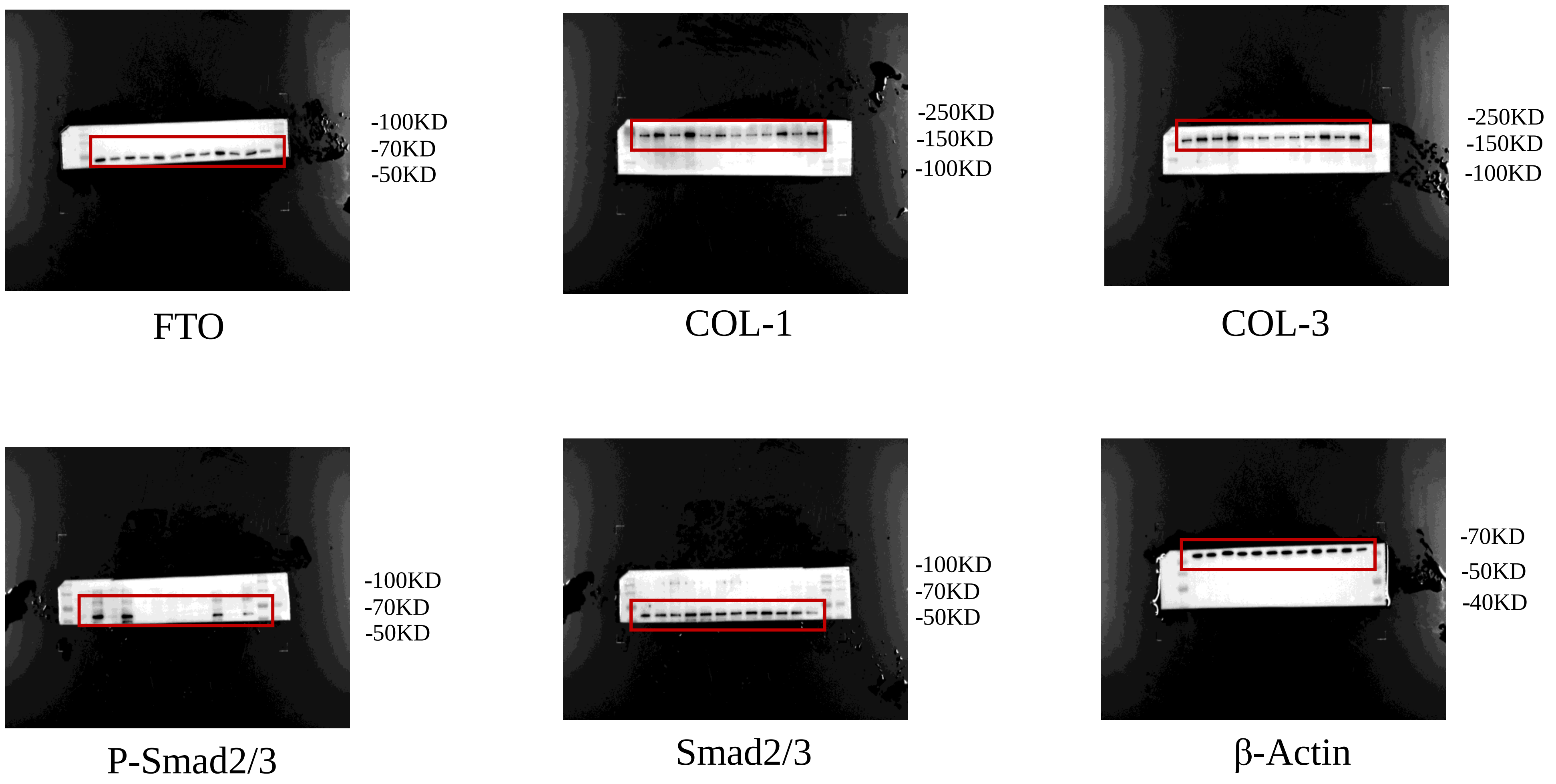
-100KD
-70KD
-50KD
FTO
-250KD
-150KD
-100KD
COL-1
-250KD
-150KD
-100KD
COL-3
-100KD
-70KD
-50KD
P-Smad2/3
-100KD
-70KD
-50KD
Smad2/3
-70KD
-50KD
-40KD
β-Actin

Figure 5 i

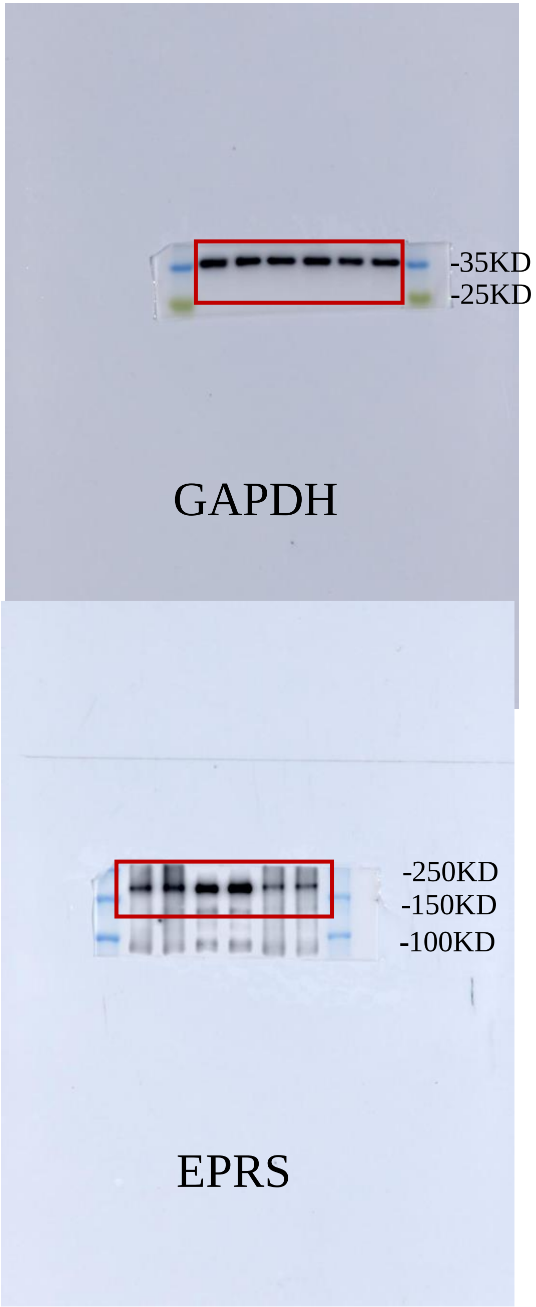

Figure 5 m

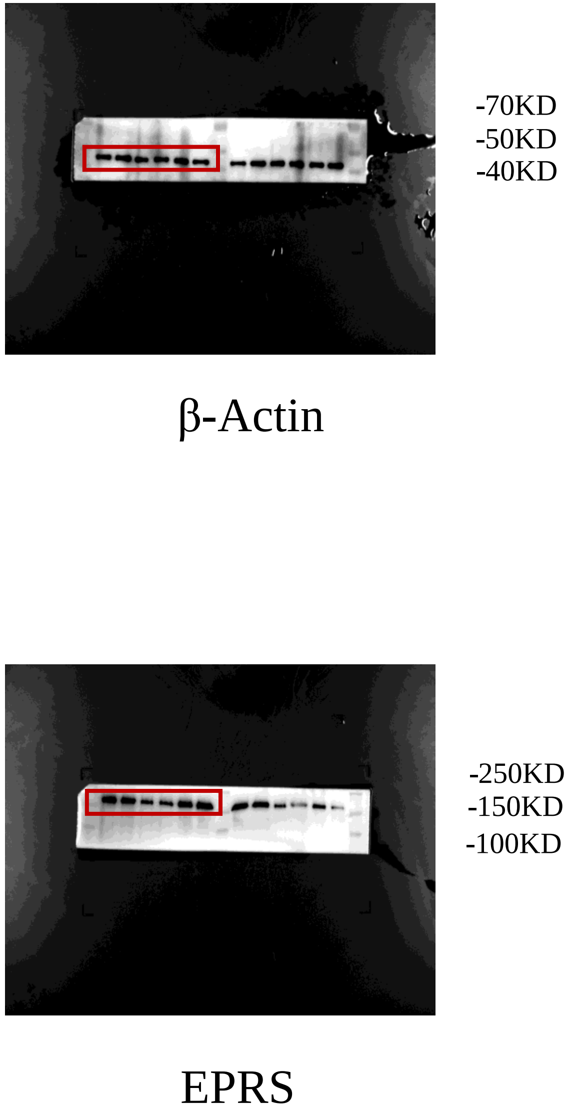

Figure 6 b

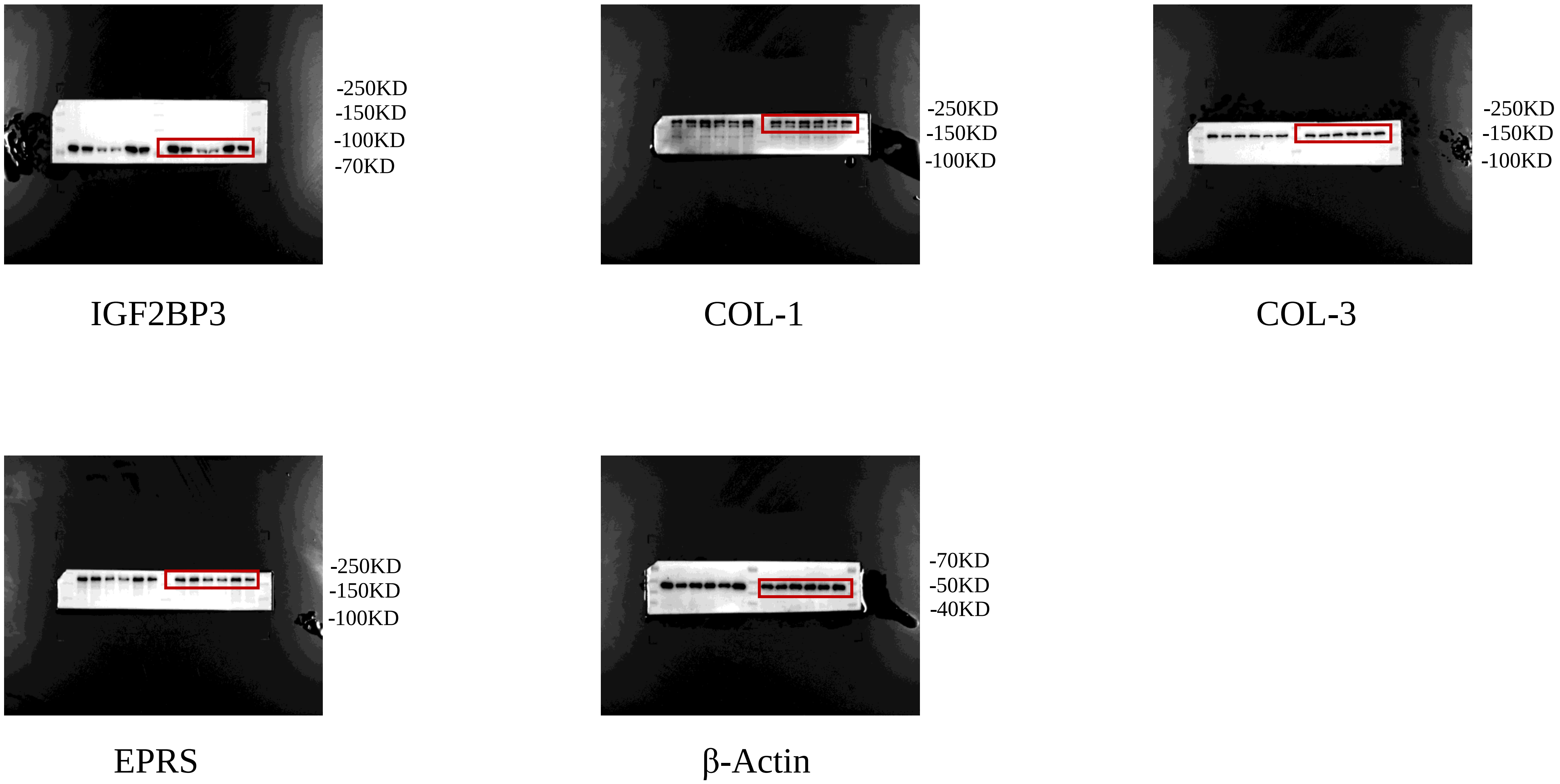
-250KD
-150KD
-100KD
-70KD
IGF2BP3
-250KD
-150KD
-100KD
COL-1
-250KD
-150KD
-100KD
COL-3
-250KD
-150KD
-100KD
EPRS
-70KD
-50KD
-40KD
β-Actin

Figure 6 c

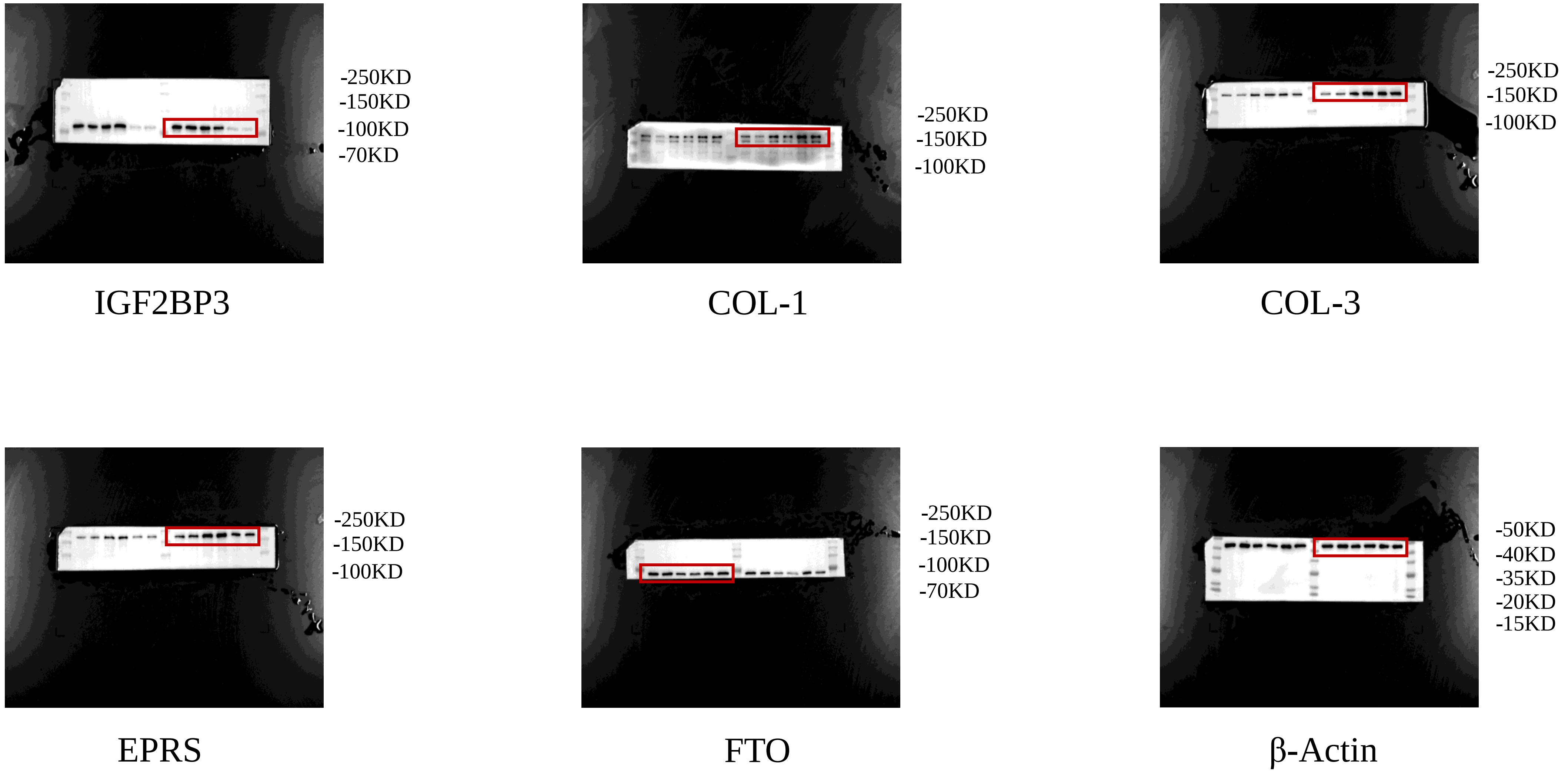
-250KD
-150KD
-100KD
-70KD
IGF2BP3
-250KD
-150KD
-100KD
COL-1
-250KD
-150KD
-100KD
COL-3
-250KD
-150KD
-100KD
EPRS
-250KD
-150KD
-100KD
-70KD
FTO
-50KD
-40KD
-35KD
-20KD
-15KD
β-Actin

Figure 7 a

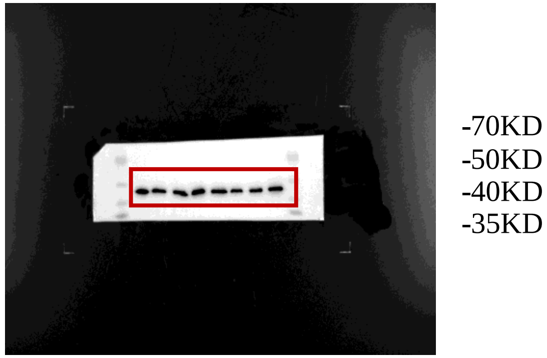

β-Actin

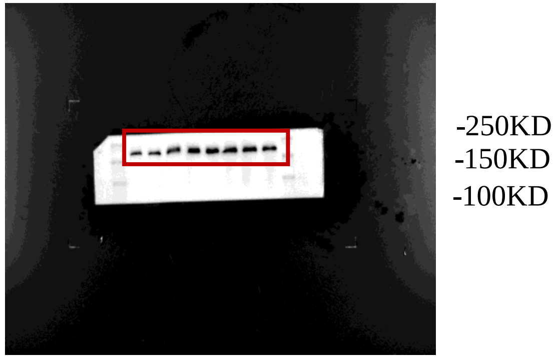

EPRS

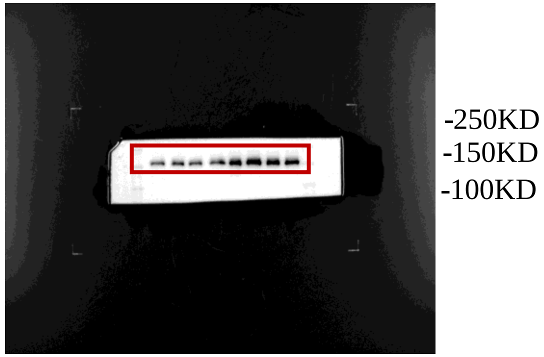

EPRS

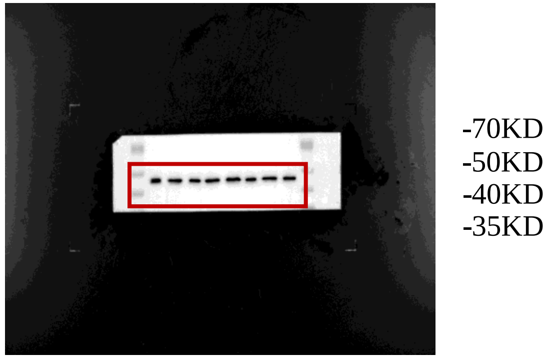

β-Actin

Figure 7 b

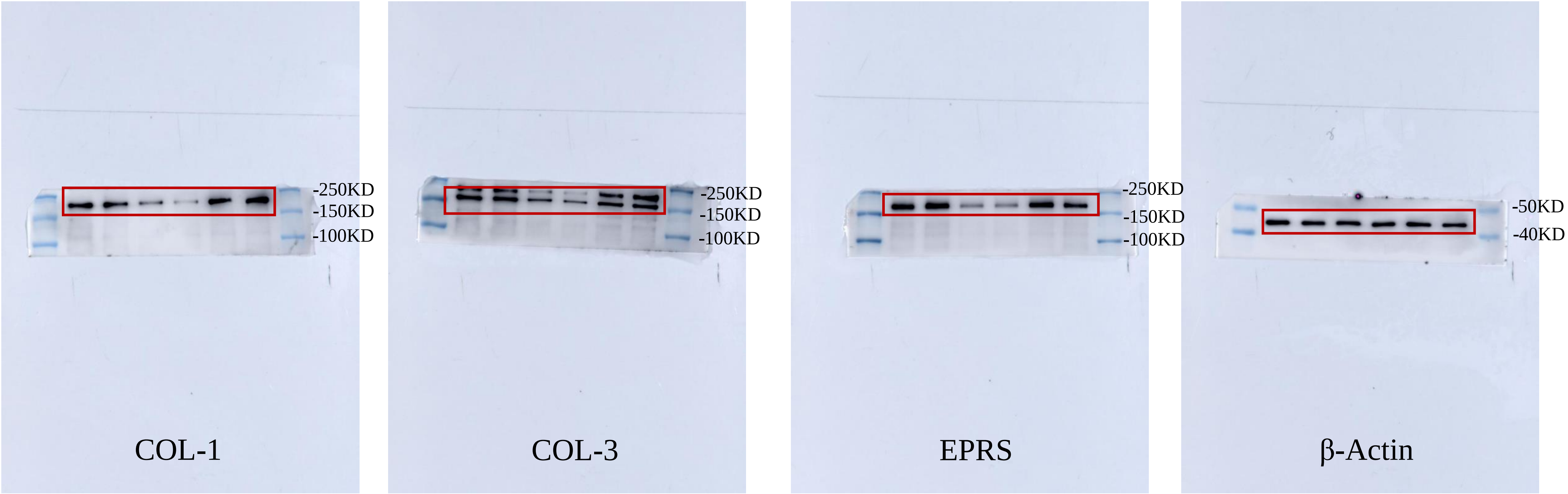
-250KD
-150KD
-100KD
COL-1
-250KD
-150KD
-100KD
COL-3
-250KD
-150KD
-100KD
EPRS
-50KD
-40KD
β-Actin

Figure 7 d

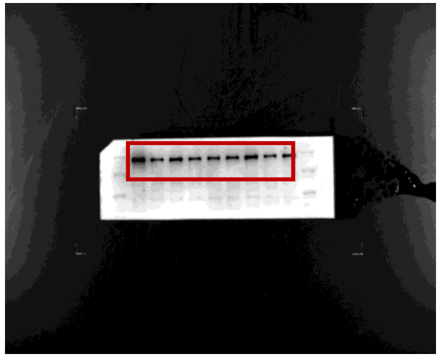

-250KD
-150KD
-100KD

COL-1

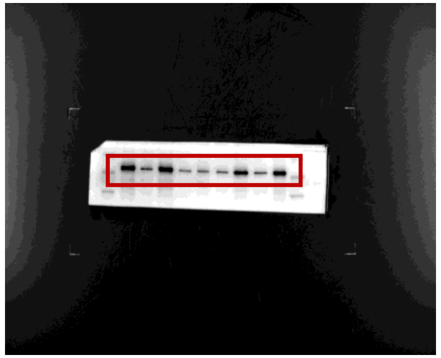

-250KD
-150KD
-100KD

COL-3

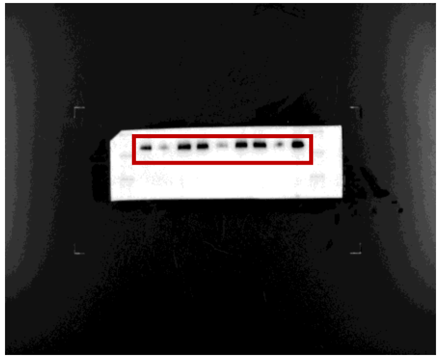

-250KD
-150KD
-100KD

EPRS

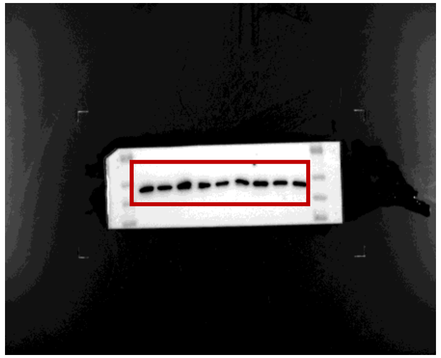

-70KD
-50KD
-40KD
-35KD

β-Actin

Figure 7 e

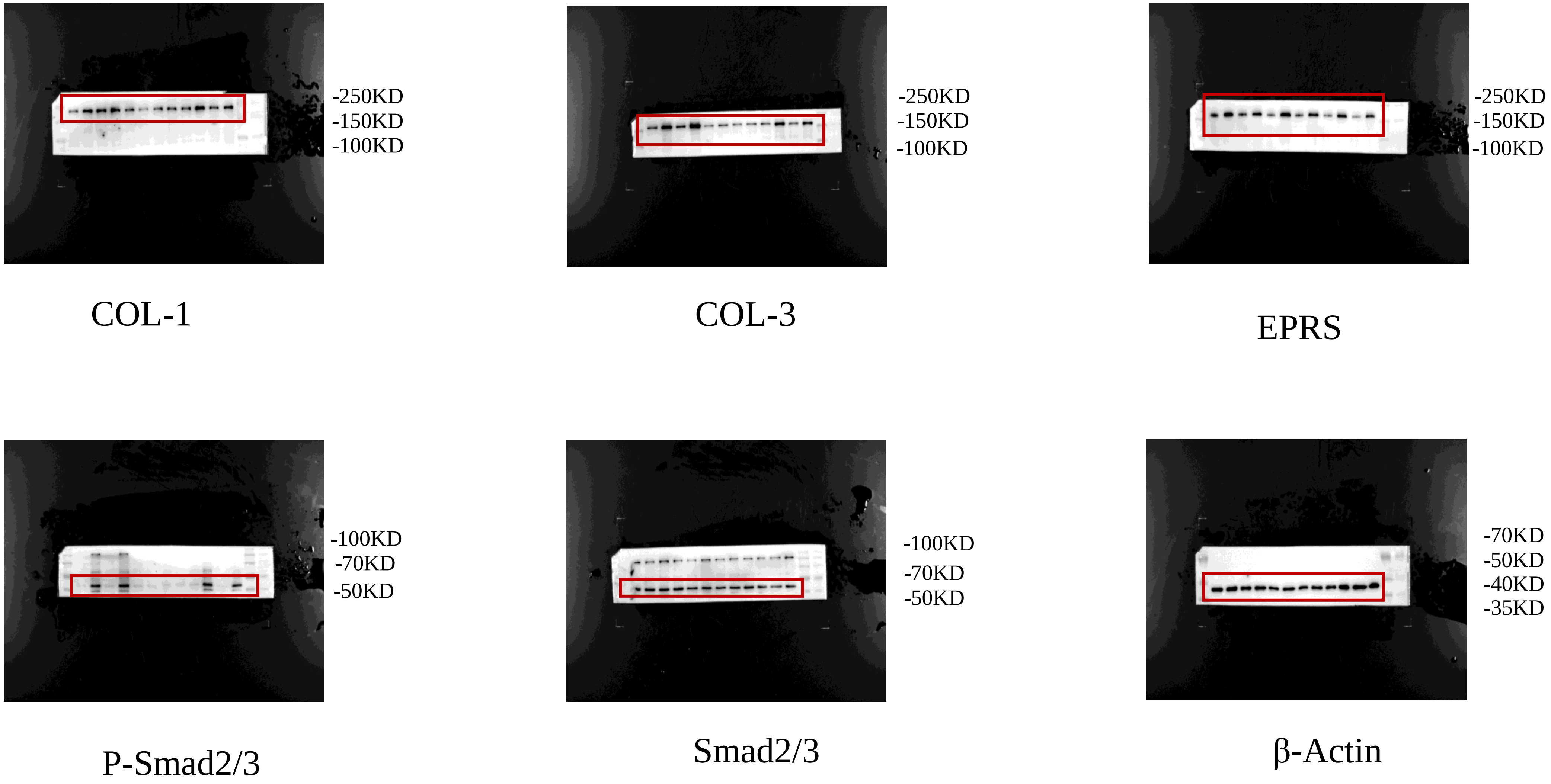
-250KD
-150KD
-100KD
COL-1
-250KD
-150KD
-100KD
COL-3
-250KD
-150KD
-100KD
EPRS
-100KD
-70KD
-50KD
P-Smad2/3
-100KD
-70KD
-50KD
Smad2/3
-70KD
-50KD
-40KD
-35KD
β-Actin

Figure 7 i

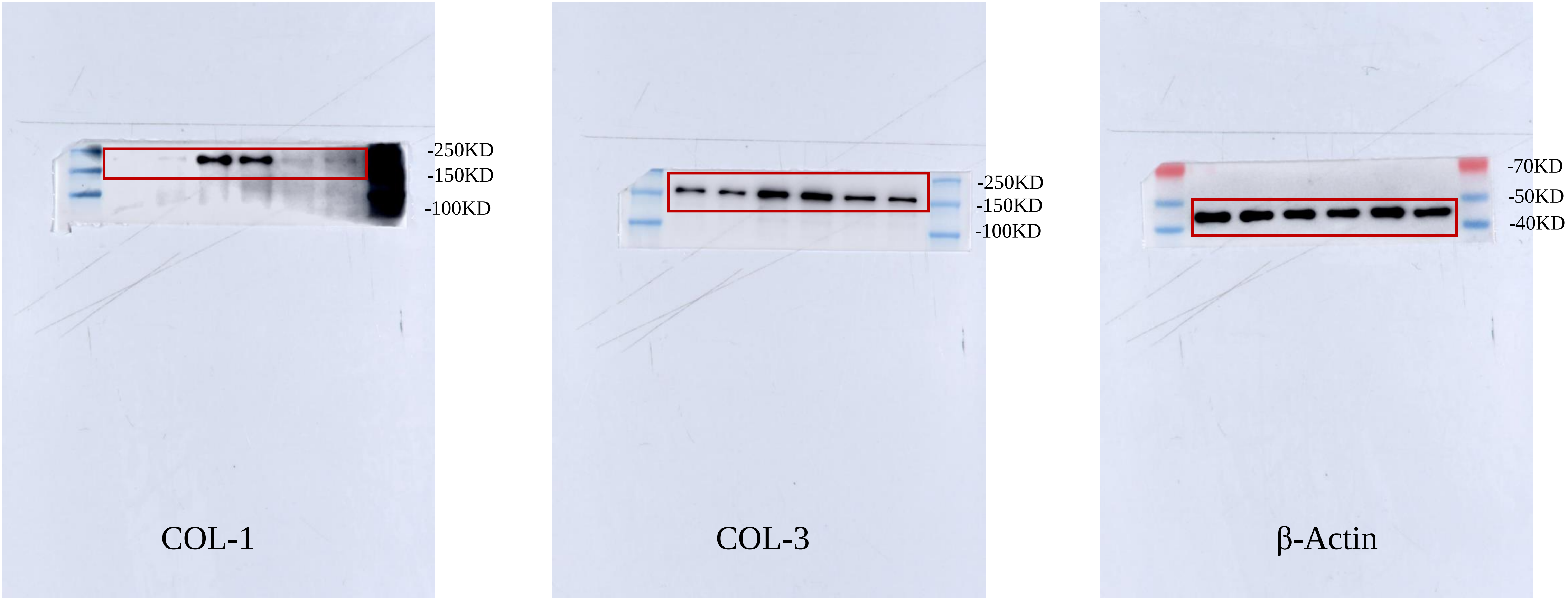
-250KD
-150KD
-100KD
COL-1
-250KD
-150KD
-100KD
COL-3
-70KD
-50KD
-40KD
β-Actin

Figure 7 k

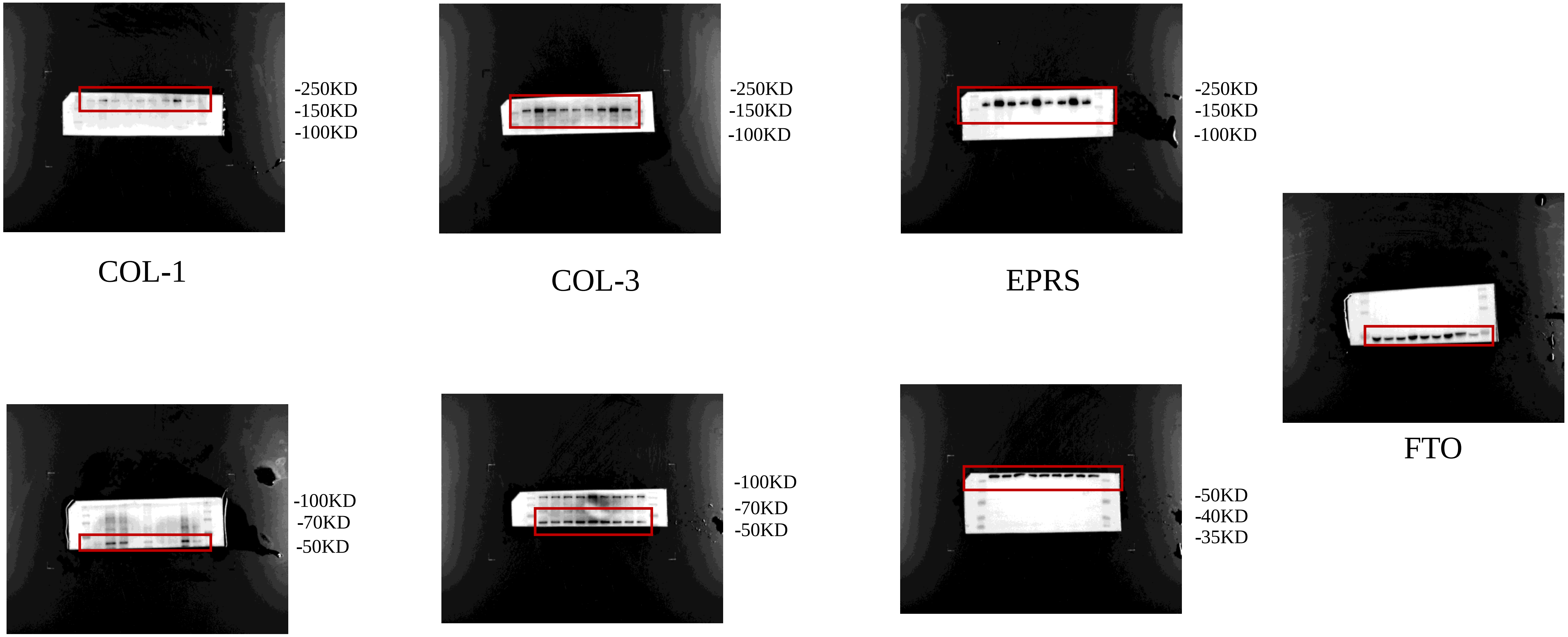

-250KD
-150KD
-100KD
COL-1
-250KD
-150KD
-100KD
COL-3
-250KD
-150KD
-100KD
EPRS
-250KD
-150KD
-100KD
-70KD
FTO
-100KD
-70KD
-50KD
P-Smad2/3
-100KD
-70KD
-50KD
Smad2/3
-50KD
-40KD
-35KD
β-Actin
